# Multifarious approaches of implementation to transfer gender sensitivity in health care practice: a scoping review

**DOI:** 10.1186/s12913-025-13032-w

**Published:** 2025-06-28

**Authors:** Judith Mollenhauer, Sophia Sgraja, Ute Seeland, Martina Kloepfer, Volker E. Amelung, Clarissa Kurscheid

**Affiliations:** 1 Institute for Epidemiology, Social Medicine and Health Systems Research, Hannover Medical School, Carl-Neuberg-Straße 1, Hanover, 30625 Germany; 2 Figus – Research Institute for Health- and System Design, Domstr. 55-73, Cologne, 50668 Germany; 3 Medical Faculty University Hospital Magdeburg Otto von Guericke, Leipziger Str. 44, Magdeburg, 39120 Germany; 4 Institute for Gender Health, Wartburgstr. 11, Berlin, 10823 Germany

**Keywords:** Gender-sensitive care, Implementation research, Implementation measures, Facilitators, Barriers, Scoping review

## Abstract

**Background:**

Gender-sensitive care (GSC+) is part of patient-centered care and necessary for adequate patient outcome. Implementation measures are needed to close the gap between evidence-based medicine and nursing and lacking gender-sensitive daily care. The present scoping review is part of the German Innovation Fund project *HeartGap* (funding number: 01VSF22030), is registered in the German Register of Clinical Studies (clinical trial registry number: DRKS 00031317, registration date: 24.02.2023), and aims to identify measures, facilitators, and barriers to enhance a sustainable implementation process of GSC+.

**Methods:**

Literature was identified from PubMed, web of science and CINAHL (2008–2023). The relevance of 23,508 articles was assessed and content analyzed. The systematic search, snowball principle, and hand search brought a total of 117 articles. The included articles were subsumed in one of the four derived main dimensions (i) politics, (ii) research/science, (iii) education, and (iv) institutions of care. The implementation of GSC+ in institutions of care (inner setting) is dependent on outer factors such as political decisions, education, or research/science (outer settings).

**Results:**

The results demonstrate facilitators and barriers, which influence the implementation of GSC+. Key measures regarding main dimensions are described as follows: (i) Politics provides the framework for the implementation of GSC+. Lobbying is a useful instrument to enhance awareness of politics. Learning from international best-practice concepts and promoting career advancement facilitates the implementation. (ii) Generating evidence by conducted studies enables the integration of GSC+ content through professional associations in guidelines. (iii) Educating (future) health care providers through GSC+ training programs, journal clubs and mandatory integration into the curricula enhances awareness of patient´s diversity. (iv) For implementing changes in daily care routine, the implementation process should be accompanied and structured by change agents. A handbook on how to organize quality circles facilitates the change process.

**Conclusion:**

The implementation of GSC+ is a cross-dimensional task. There are already many measures that can sustainably improve gender sensitivity in the dimensions. There is still a lack of compulsory requirements for implementation and recommended actions to ensure that GSC+ is considered in practice.

**Supplementary Information:**

The online version contains supplementary material available at 10.1186/s12913-025-13032-w.

## Introduction

Evidence-based medicine (EBM) and evidence-based nursing (EBN) of gender-sensitive care (GSC+) are considered in medical guidelines and nursing expert standards and are the basis for qualitative-based patient care [1, 2]. Translating EBM and EBN in daily practice requires implementation strategies on several dimensions. Implementation science aims to identify facilitators, barriers, and measures to achieve successful implementation. Established theories and frameworks, such as Rogers´ Model of Five Stages in the Innovation Process or the Five Stages in the Innovation Process in Organizations [3], or the Updated Consolidated Framework for Implementation Research (CFIR) [4] of implementation science states established dimensions that are to be considered in the implementation process. That includes individuals, the innovation, the implementation process, the inner setting (organization within individuals), and the outer setting (community, system, state) [3, 4], which are similar to those of the identified and established dimensions of the GSC+ by Oertelt-Prigione in her habilitation paper. Oertelt-Prigione enumerates outer and inner settings, which are relevant to consider while implementing GSC+ in practice. These are hospitals (inner setting), universities, professional associations, institutes of science, promoter of science, health insurances, policies, media and general population (outer settings) [5].

Women and men require different medical and nursing care due to organical, hormonal and behavioral conditions and risk factor differences. Therefore, equal treatment of women and men in health care can lead to inadequate medical care [6]. GSC+ requires inclusion of gender in classifying of symptoms and awareness in diagnostics, therapy, and nursing care. GSC+ means a qualitative and patient-orientated care, where biological and social differences between women and men are taken in consideration [7]. It involves the different perceptions on health and illness, uptake of preventive measure and drug compatibility of female and male bodies´ [8]. Medical guidelines and nursing expert standards include content on how to care gender-sensitive, but the degree of implementation due to professionals in health care practice in Germany is unknown. There are indicators that GSC+ is not implemented adequately. Within the framework of the project “HeartGap” – funded from the German Federal Joint Committee (funding number: 01VSF22030) – the hypothesis will be investigated based on patients with acute and chronic myocardial syndromes [9]. Particularly, it is surveyed that men suffer more frequently myocardial infarctions, but women’s rate of lethality is higher in comparison to men’s [10]. Hence, it is important to identify relevant measures of implementation and influencing factors to reduce inequalities and different outcomes in patient care. The implementation of GSC+ in clinics is dependent on outer factors as political decisions or social-cultural environment, and inner organizational factors, such as time pressure or organizational culture.

The aim of the scoping review is to give an overview about relevant measures for hospitals and their professionals (inner setting) and outer settings to implement or enhance GSC+. Facilitating factors, measures and obstacles are identified for implementation process support.

## Methods

This scoping review follows the recommendations of cochrance 2019 and the Preferred Reporting Items for Systematic reviews and Meta-Analyses extension for Scoping Review (Prisma ScR) [11, 12]. The multifariousness and the range of measures of different evidence and dimension levels are shown by the authors with the method of a scoping literature review.

The databases Pubmed, web of science and CINAHL were used to identify relevant articles. All search results cover fifteen years (2008–2023) and are composed in German or English language. The total searching period was from 06/07/2023 to 15/11/2023. This time period was selected to follow up on the results of the systematic review by Celik et al. who conducted a content analysis of the literature on the implementation of gender sensitivity in health care [13]. Several keywords were used to cover the previously identified dimensions and were connected with Boolean operators. An individual search term was used for each dimension and literature database (Table 1). For more precise search results a search term was created that did not exceed more than 1,000 title results.

**Table 1:** Search terms for each established dimension

Dimension	Search term
Hospitals (Inner Setting)	(woman[Title/Abstract] OR women[Title/Abstract] OR sex[Title/Abstract] OR female[Title/Abstract] OR man[Title/Abstract] OR men[Title/Abstract] OR male[Title/Abstract] OR transgender[Title/Abstract]) AND (consultation[Title/Abstract] OR “consultation hour“[Title/Abstract] OR clinic[Title/Abstract] OR guidance[Title/Abstract] OR hospital[Title/Abstract] OR counsel[Title/Abstract]) AND (“gender sensitivity“[Title/Abstract] OR “gender medicine“[Title/Abstract] OR “gender specific care“[Title/Abstract] OR “gender sensibility“[Title/Abstract] OR “gender awareness“[Title/Abstract]) AND (2008:2023[pdat])
Health care professionals (Inner setting)	(woman[Title/Abstract] OR women[Title/Abstract] OR sex[Title/Abstract] OR female[Title/Abstract] OR man[Title/Abstract] OR men[Title/Abstract] OR male[Title/Abstract] OR transgender[Title/Abstract]) AND (nurse[Title/Abstract] OR nursing[Title/Abstract] OR nurs*[Title/Abstract] OR physician[Title/Abstract] OR doctor[Title/Abstract] OR arzt[Title/Abstract] OR cardiology[Title/Abstract] OR cardio*[Title/Abstract] OR “health profession“[Title/Abstract]) AND (“gender sensitivity“[Title/Abstract] OR “gender medicine“[Title/Abstract] OR “gender specific care“[Title/Abstract] OR “gender sensibility“[Title/Abstract] OR “gender awareness“[Title/Abstract]) AND (2008:2023[pdat])
Universities (Outer Setting)	(woman[Title/Abstract] OR women[Title/Abstract] OR sex[Title/Abstract] OR female[Title/Abstract] OR man[Title/Abstract] OR men[Title/Abstract] OR male[Title/Abstract] OR transgender[Title/Abstract]) AND (education[Title/Abstract] OR curriculum[Title/Abstract] OR training[Title/Abstract] OR teach[Title/Abstract] OR university[Title/Abstract]) AND (“gender sensitivity“[Title/Abstract] OR “gender medicine“[Title/Abstract] OR “gender specific care“[Title/Abstract] OR “gender sensibility“[Title/Abstract] OR “gender awareness“[Title/Abstract]) AND (2008:2023[pdat])
Professional association (Outer setting)	(woman[Title/Abstract] OR women[Title/Abstract] OR sex[Title/Abstract] OR female[Title/Abstract] OR man[Title/Abstract] OR men[Title/Abstract] OR male[Title/Abstract] OR transgender[Title/Abstract]) AND (“professional association“[Title/Abstract] OR “professional society“[Title/Abstract] OR “task force” [Title/Abstract] OR “work group“[Title/Abstract] OR corporation[Title/Abstract] OR workforce[Title/Abstract]) AND (“gender sensitivity“[Title/Abstract] OR “gender medicine“[Title/Abstract] OR “gender specific care“[Title/Abstract] OR “gender sensibility“[Title/Abstract] OR “gender awareness“[Title/Abstract]) AND (2008:2023[pdat])
Research institutes (Outer setting)	(woman[Title/Abstract] OR women[Title/Abstract] OR sex[Title/Abstract] OR female[Title/Abstract] OR man[Title/Abstract] OR men[Title/Abstract] OR male[Title/Abstract] OR transgender[Title/Abstract]) AND (“research institute“[Title/Abstract] OR “gender research“[Title/Abstract] OR survey[Title/Abstract] OR trial[Title/Abstract]) AND (“gender sensitivity“[Title/Abstract] OR “gender medicine“[Title/Abstract] OR “gender specific care“[Title/Abstract] OR “gender sensibility“[Title/Abstract] OR “gender awareness“[Title/Abstract]) AND (2008:2023[pdat])
Promoter of science (Outer setting)	(woman[Title/Abstract] OR women[Title/Abstract] OR sex[Title/Abstract] OR female[Title/Abstract] OR man[Title/Abstract] OR men[Title/Abstract] OR male[Title/Abstract] OR transgender[Title/Abstract]) AND (“main research“[Title/Abstract] OR “research concentration“[Title/Abstract] OR “research project“[Title/Abstract] OR “research focus“[Title/Abstract]) AND (“gender sensitivity“[Title/Abstract] OR “gender medicine“[Title/Abstract] OR “gender specific care“[Title/Abstract] OR “gender sensibility“[Title/Abstract] OR “gender awareness“[Title/Abstract]) AND (2008:2023[pdat])
Health insurance (Outer setting)	(woman[Title/Abstract] OR women[Title/Abstract] OR sex[Title/Abstract] OR female[Title/Abstract] OR man[Title/Abstract] OR men[Title/Abstract] OR male[Title/Abstract] OR transgender[Title/Abstract]) AND (insurance[Title/Abstract] OR payer[Title/Abstract] OR provider[Title/Abstract]) AND (“gender sensitivity“[Title/Abstract] OR “gender medicine“[Title/Abstract] OR “gender specific care“[Title/Abstract] OR “gender sensibility“[Title/Abstract] OR “gender awareness“[Title/Abstract]) AND (2008:2023[pdat])
Politics (Outer setting)	(woman[Title/Abstract] OR women[Title/Abstract] OR sex[Title/Abstract] OR female[Title/Abstract] OR man[Title/Abstract] OR men[Title/Abstract] OR male[Title/Abstract] OR transgender[Title/Abstract]) AND (politics[Title/Abstract] OR stakeholder[Title/Abstract]) AND (“gender sensitivity“[Title/Abstract] OR “gender medicine“[Title/Abstract] OR “gender specific care“[Title/Abstract] OR “gender sensibility“[Title/Abstract] OR “gender awareness“[Title/Abstract]) AND (2008:2023[pdat])
Media (Outer setting)	(woman[Title/Abstract] OR women[Title/Abstract] OR sex[Title/Abstract] OR female[Title/Abstract] OR man[Title/Abstract] OR men[Title/Abstract] OR male[Title/Abstract] OR transgender[Title/Abstract]) AND (experts[Title/Abstract] OR media[Title/Abstract] OR “new media“[Title/Abstract] OR journalists[Title/Abstract]) AND (“gender sensitivity“[Title/Abstract] OR “gender medicine“[Title/Abstract] OR “gender specific care“[Title/Abstract] OR “gender sensibility“[Title/Abstract] OR “gender awareness“[Title/Abstract]) AND (2008:2023[pdat])
Population (Outer setting)	(woman[Title/Abstract] OR women[Title/Abstract] OR sex[Title/Abstract] OR female[Title/Abstract] OR man[Title/Abstract] OR men[Title/Abstract] OR male[Title/Abstract] OR transgender[Title/Abstract]) AND (population[Title/Abstract] OR society[Title/Abstract] OR public[Title/Abstract]) AND (“gender sensitivity“[Title/Abstract] OR “gender medicine“[Title/Abstract] OR “gender specific care“[Title/Abstract] OR “gender sensibility“[Title/Abstract] OR “gender awareness“[Title/Abstract]) AND (2008:2023[pdat])
Health care professionals (Inner setting)	(woman[Title/Abstract] OR women[Title/Abstract] OR sex[Title/Abstract] OR female[Title/Abstract] OR man[Title/Abstract] OR men[Title/Abstract] OR male[Title/Abstract] OR transgender[Title/Abstract]) AND (nurse[Title/Abstract] OR nursing[Title/Abstract] OR nurs*[Title/Abstract] OR physician[Title/Abstract] OR doctor[Title/Abstract] OR arzt[Title/Abstract] OR cardiology[Title/Abstract] OR cardio*[Title/Abstract] OR “health profession“[Title/Abstract]) AND (“gender sensitivity“[Title/Abstract] OR “gender medicine“[Title/Abstract] OR “gender specific care“[Title/Abstract] OR “gender sensibility“[Title/Abstract] OR “gender awareness“[Title/Abstract]) AND (2008:2023[pdat])
General search term (GSC+ in cardiology)	(woman[Title/Abstract] OR women[Title/Abstract] OR sex[Title/Abstract] OR female[Title/Abstract] OR man[Title/Abstract] OR men[Title/Abstract] OR male[Title/Abstract] OR transgender[Title/Abstract]) AND cardio*[Title/Abstract] AND (“gender sensitivity“[Title/Abstract] OR “gender medicine“[Title/Abstract] OR “gender specific care“[Title/Abstract] OR “gender sensibility“[Title/Abstract] OR “gender awareness“[Title/Abstract]) AND (2008:2023[pdat])
General search term (implementation of GSC+)	(woman[Title/Abstract] OR women[Title/Abstract] OR sex[Title/Abstract] OR female[Title/Abstract] OR man[Title/Abstract] OR men[Title/Abstract] OR male[Title/Abstract] OR transgender[Title/Abstract]) AND implementation[Title/Abstract] AND (“gender sensitivity“[Title/Abstract] OR “gender medicine“[Title/Abstract] OR “gender specific care“[Title/Abstract] OR “gender sensibility“[Title/Abstract] OR “gender awareness“[Title/Abstract]) AND (2008:2023[pdat])
	(measure* OR obstacles OR facilitating factors) AND (implementation) AND (gender sensitiv* OR gender medicine OR gender specific area OR gender awareness) AND (2008:2023[pdat])

The results of the systematic literature search were extended by a snowball principle and hand search. For this purpose, the references of relevant and included articles were screened with backward snowballing and forward snowballing (identifying subsequent citations using Google Scholar). The same assessment procedure with title, abstract, and full-text screening was conducted. To identify grey literature, we used the search engines Google Scholar and Google. Various combinations of our general search terms were applied to ensure broad coverage. Titles and content of the search results were screened systematically. In addition, relevant grey literature was identified through hand searches on websites of key institutions and relevant portals. Sources were selected based on their relevance to the research question, inclusion criteria, and the reviewers’ informed judgment. The relevance for the inclusion of each article was assessed according to the following inclusion criteria:

i) Articles that address the topic of GSC+ and/or transgender/LGBTQ + health.
ii) Articles that include facilitating factors and/or barriers and/or measures regarding the implementation of GSC+.
iii) German or English language.
iv) Studies published between 2008 and 2023.

The exclusion criteria for the articles were as follows:

i) Articles that contain only measures or barriers related to career advancement in health care.
ii) Articles, which do not have a direct reference to GSC+.
iii) Articles that have a review design.

Two reviewers independently reviewed the titles and abstracts to determine eligibility for full-text review. In the initial search, a total of 23,508 titles were found. After identification of articles, titles, abstracts, and full-text articles were screened. Articles were excluded, if they did not meet the previously determined inclusion criteria. Furthermore, 291 duplicates were identified and excluded. Subsequently, 340 abstracts were checked. Within those 340 abstracts 133 articles were eliminated due to lack of relevance and previously determined language (German or English).

**Fig. 1:**
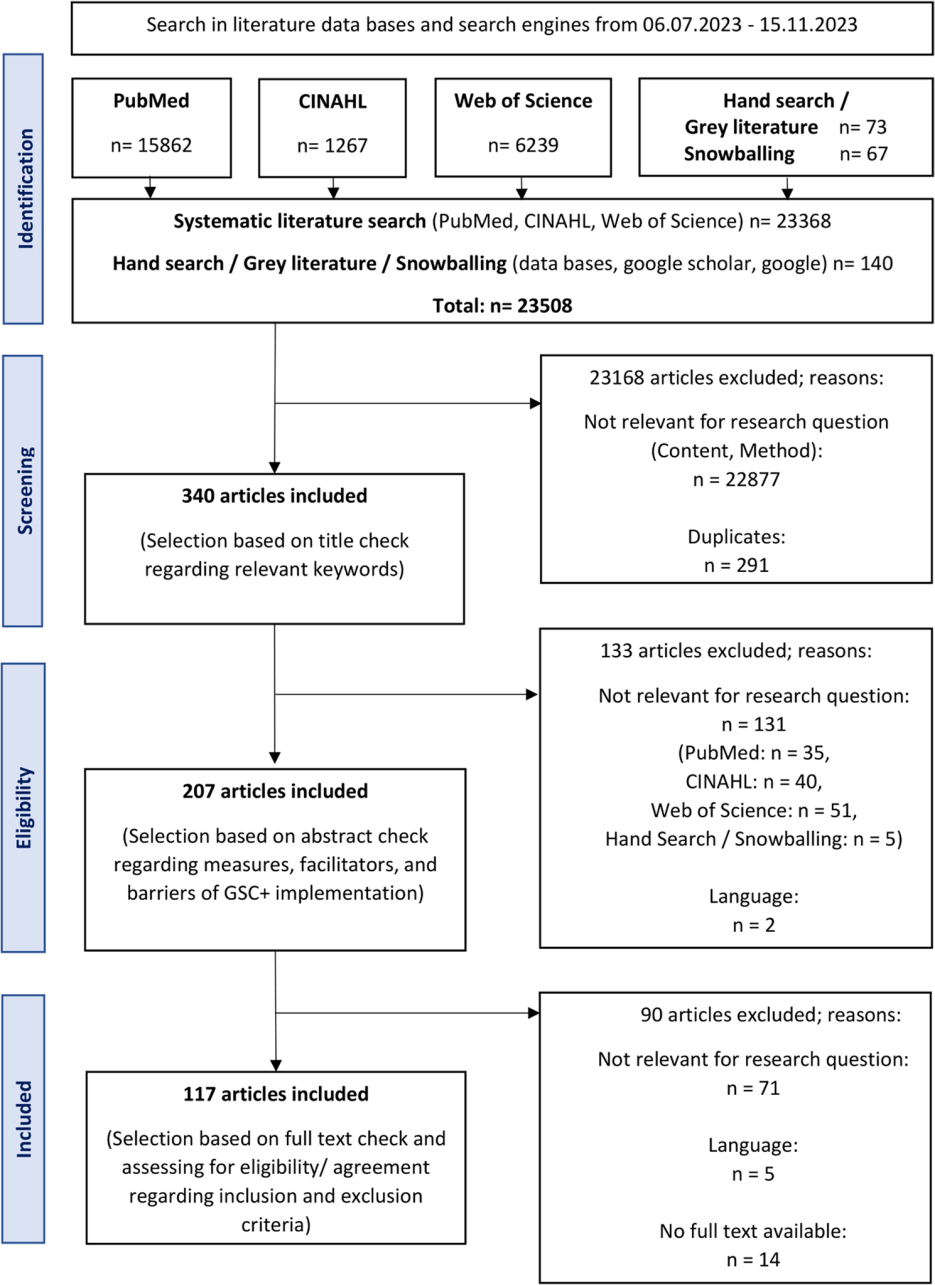
PRISMA flow diagram of the study selection process

In total, 207 full-text articles were analyzed in regard to answer the research question. The articles were discussed by the two reviewers and agreements were made on inclusion or exclusion of the articles. There were a few inconsistencies in the decisions to include or reject articles. In the case of disagreements, the two reviewers discussed the case on the basis of the explained inclusion and exclusion criteria and came to an agreement. Ninety ineligible articles (content and language) and unavailable articles were excluded. The remaining 117 articles (116 scientific articles and 1 grey literature source [14]) were scanned for the following categories: Facilitating measures/facilitators and barriers. Following, these contents were collected in a table subdivided into these two categories. The systematic search, snowball principle and hand search brought a total of 117 articles (Fig. 1). The included articles were subsumed under an adequate main and sub-dimension. While certain dimensions were adopted from the existing frameworks, four main dimensions – “Education”, “Research/science”, “Institutions of care”, and “Politics” – were additionally derived as part of the analysis. In the Supplementary Material 1: Appendix “Overview of results”, there is a table with an overview of all included articles. Overall, the level of evidence ranges between II and V. The type of studies included varies and ranges from expert opinions/comments to RCTs. The focus of this scoping review was to identify the content of the GSC+ implementation measures, facilitators, and barriers. The study and quality assessment procedure for the present scoping review is based on determining the level of evidence and reviewers´ assessment of methodological and content consistency, especially the research question to applied methodology. In conclusion, no articles were excluded due to content or methodological reasons.

## Results

### Characteristics of the study

**Fig. 2:**
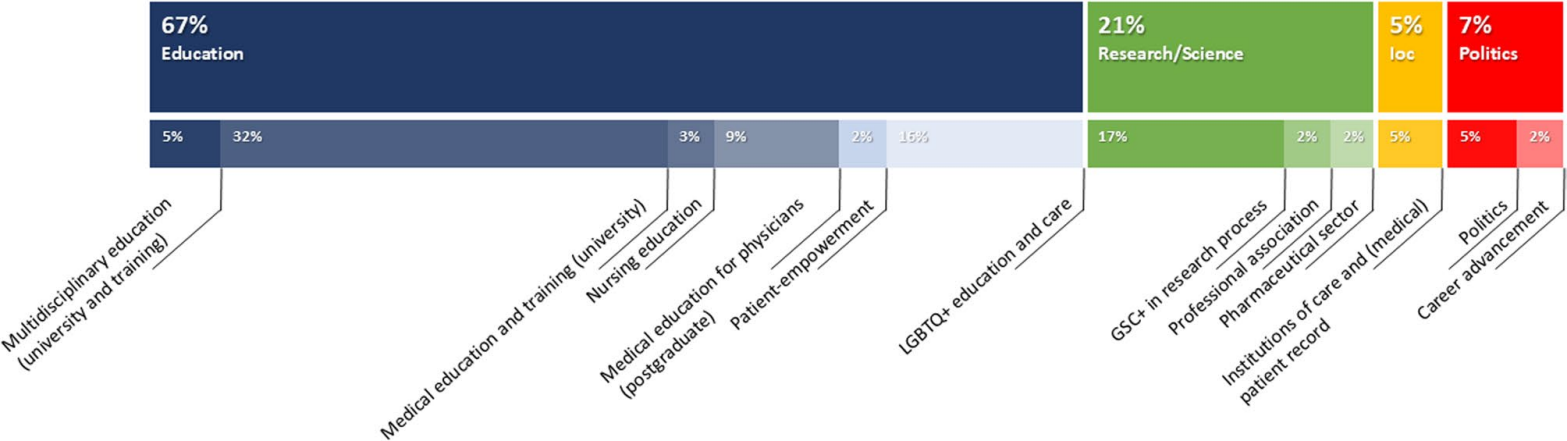
Overview of dimensions (Upper bar chart: Main dimensions; Bar chart below: Subsumed dimensions with proportions of the included articles). Abbreviation: Ioc = Institutions of care

Figure 2 shows the four derived main dimensions “Education”, “Research/science”, “Institutions of care”, and “Politics” in the upper bar chart and the subsumed dimensions of education in blue (*n* = 6), dimensions of research/science in green (*n* = 3), dimension of institutions of care (Ioc) in yellow (*n* = 1), and dimensions of politics in red (*n* = 2) in the bar chart below. The different shades of blue, green, yellow, and red were used for better differentiation and have no further meaning. In total four derived main dimensions and 12 subdimensions were identified. Around two-thirds of the identified dimensions are found in the main dimension of education (67 %).

### Measures, facilitators, and barriers of implementation

The contents from the analyzed articles are presented by derived main dimensions in the following result sections.

**Fig. 3:**
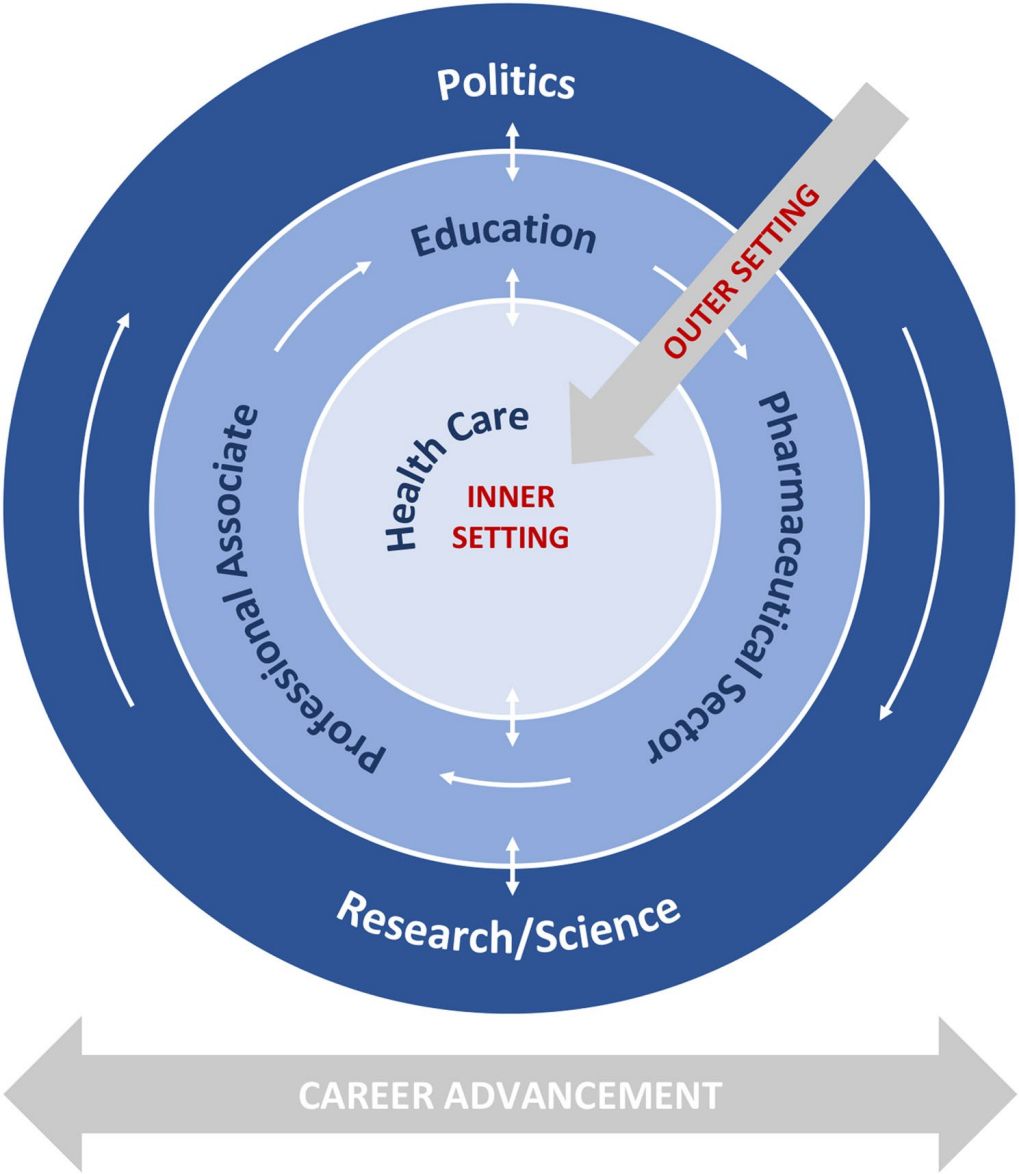
Bull´s eye diagram. Dimensions of promoting GSC+ in health care practice (inner and outer setting)

The implementation of GSC+ in health care practice is influenced by outer setting (Fig. 3).

Politics provides the framework for the implementation of GSC+ and interrelates research and science regarding gender sensitivity. Both influence the degree of promoting gender sensitivity in education, professional associations and guideline development as well as for pharmaceutical sector and gender-sensitive drug development. The implementation intensity in the health care institutions depends on previously mentioned levels. Career advancement can promote GSC+ in all dimensions.

#### Main dimension – politics

By enacting legislation, the government of a state supports topics that would remain less supported by population if they were not anchored in law. Thus, a topic receives more attention, support, and funding. For setting an agenda point, lobbying is a useful instrument to enhance awareness in politics. The more federal states are consent about certain agenda points, the greater the willingness becomes to draft laws and implement them. Politics consults stakeholders and experts in form of workshops for developing overarching strategies and principles [15].

Currently, different strategies are described to implement GSC+ in politics. In general, analyzing and identifying gaps in GSC+ are essential for developing tailored strategies [16, 17]. Gagliardi et al. described, that equipping policy-makers with tools or providing education facilitates development of GSC+-informed policies [17]. In contrast, factors that impede sustainable and successful implementation of GSC+ are lack of political resources, misallocation of budgets and political instability [16].

Funded projects of political measures from Canada, USA and Germany are described below:

In Canada a National Institute of Gender and Health was founded with the aim to facilitate gender equity and health research. The institute consults the politics in agenda setting the research priority, provide adequate instruments, setting up arrangements of research capacities and translate research results to health care practice and politics. The institute is financed by public finances, which demonstrates the political interest und support in promoting the topic GSC+ in Canada [18].

In the USA, the National Institutes of Health and the U.S. Food and Drug Administration are responsible for implementing and funding sex and gender in biomedical and drug and medical devices research. “[…] new U.S. federal laws for the practice of research, the development of federally-funded studies on the health of women, and the establishment of interdisciplinary research collaborations and centers at academic health centers” and the assignment to use adequate methods for gender-sensitive health information are parts of the Strategic Plan on political level to promote requirements of the implementation regard to GSC+ [18].

In Germany the federal state North Rhine-Westphalia initiated the integration of a gender-differentiated perspective into health policy and health care. The implementation strategy was built on four pillars between 2000 and 2006: (1) women’s policy resolutions and initiatives, (2) a gender-differentiated orientation of health policy, (3) the introduction of gender mainstreaming as a guiding principle of government action and (4) the development of supporting structures at state level. For the implementation following political measures were applied: (1. pillar) Action weeks in 150 cities with information events for women, telephone consultation hours on health issues, specialist conferences, health conference on women and health, (2. pillar) involvement of the state parliament in the commission of inquiry, annual scientific health report on various target groups, legal inclusion of women in clinical studies, introduction of gender as a category in state-wide health policy, systematic consideration of gender aspects in the planning and review of projects, (3. pillar) introduction of equal opportunities officers, gender-education for senior management, (4. pillar) gender matrix as a process-oriented instrument for considering the gender perspective in the health sector (gender equality goal, sex/gender, theories, concepts, methods/instruments, participation) and working groups on gender aspects of health care [19].

Politicians are responsible to reflect GSC+ at all stages of policy making and program development and to ensure that all cooperation partners implement measures in health politics [20, 21]. In particular, for the last years increased attention has been given to the topics of diversity, equity and inclusion, including health care. Lesbian, gay, bisexual, transgender and queer+ (LGBTQ+) people and people of color can be affected by disrespect and discrimination, leading to inadequate treatment or difficulties in accessing appropriate healthcare. Goldberg et al. suggest improving healthcare access for LGBTQ + people through more protective policies [22].

##### Career advancement

Predominantly more women than men work in the healthcare system (75% female employee, 25% male employee), especially in nursing (82% female nurses, 18% male nurses in Germany 2023) [23, 24]. It is noticeable, that male health care professionals are more often in leadership positions than women. Studies have shown that women in leadership positions in research, teaching and healthcare are leading to positive effects and advancement in healthcare quality [25–28]. As shown in Fig. 3, career advancement is a cross-dimensional dimension.

On research and science level, Gagliardi recommended engaging women on guideline-writing panels to consider several perspectives in guideline development [25]. Overall, there should be a balanced gender ratio among researchers to achieve gender awareness in research [26].

On the educational level, mentoring programs can help promoting women to achieve leadership positions. Further, implementation of endowed chairs in women’s health facilitate “women’s leadership, the institutional stature of women’s health, and activities in women’s health research, education, and clinical care” [27].

On health care level, female managers could enrich the leadership culture in medicine. An increased number of female managers could have a positive effect on focused advancement of gender medicine. Finding the balance between female and male managers in health care institutions should be pursued. In context of patient care, Oldhafer stated that on average female doctors see their role as a companion or supporter and promote the compliance through intensive exchanges with patients in comparison to male doctors. In addition to the health aspects, GSC+ has financial benefits. In conclusion, patients that are treated gender-specifically early on, were noticed to have fewer and shorter sick days and lower costs were generated [28].

#### Main dimension – research and science

##### GSC+ in research process

In addition to politics, research and the resulting evidence-based medicine and Nursing of GSC+ are the basis and essential for implementation in health care practice. In the last years the consideration of GSC+ in research funding has become more relevant. For instance the integration of GSC+ in studies and research projects are compulsive by research funders [29]. Application forms for funding announcement require GSC+ criteria inclusion [30]. To create an incentive for researchers to implement dimensions of sex and gender in research, explicit GSC+ education is offered. Relevant aspects of gender occurs in all specialties and should be considered in all phases of research, from biomedical and clinical to epidemiological and public health research [31–33]. Further, it is highly important for subsequent research steps (decision of funding, project objective, research questions, methods, data analysis, evaluation) [34]. In addition to researchers, reviewers and committee members need to be educated to enhance their sensibilization and awareness of gender sensitivity. Tools, such as the online CIHR (considerations into health research) modules and virtual education or in-depth local training programs, such as workshops, personal training, educational and research initiatives in form of formal networks and summer schools are conceivable [26, 29, 30, 35].

Guidelines for research and project designing support researchers while realization of gender-sensitive research [36]. For instance, the SIMI Gender ‘5 Ws’ Rule (**W**ho (Clinical Internal Medicine Scientists and Practitioners), **W**hat (Gender-related Variables—Gender Core Dataset), **W**here (Clinical Studies/Translational Research), **W**hen (Every Time It Makes Sense) and **W**hy (Explanatory Power of Gender and Opportunities)) is a conceptual framework that guides researchers of internal medicine by designing and analyzing clinical studies [37] or the gender lens tool, which supports the implementation of sex and gender in researchers´ work. The gender lens tool provides gender-specific questions of biological, social, economic, political and educational factors and of incidence/prevalence, diagnostic/investigations, risk factors, natural history and treatment to consider gender sensitivity at each step of project designing or research process [38].

Two relevant tools are known to facilitate the literature searching process. The Texas Tech University Health Science Center Sex and gender specific health PubMed Search Tool (TTUHSC) helps researchers to find articles on PubMed data base with focus on sex and gender. The TTUHSC expanded ((sex based OR sex factors OR sex distribution OR sex characteristics OR sex dimorphism OR gender difference* OR female) AND (gender[ti] OR sex[ti] OR women[ti] OR female[ti]); filters: human, English) can be connected with further key words to specify the search results [39]. Further, the GenderMedDB is an interactive data base for gender-specific medical literature, which retrieves literature from PubMed database and offers the advantages of a gender medicine specific search engine [40].

Building of interdisciplinary educated research teams with a balanced gender ratio enhance a diversity awareness and perspective [26, 41, 42]. Calls for tender of Horizon Europe (EU’s key funding program for research and innovation) enable explicit to budget resources in research projects for acquirement gender competence [29].

In addition to a gender-sensitive research process, gender-sensitive data analysis and reporting is to be considered by researchers, authors and publisher [43]. Four steps are essential for gender-sensitive reporting: (1) gender comparative analysis, (2) gender differentiated analysis, (3) consideration of frameworks of society and (4) classification of the results based on theoretical explanations [44]. For standardizing gender-sensitive reporting in publications, the Sex and Gender Equity in Research (SAGER)-guideline should be used by researchers and authors. Appropriate use of terminology (sex versus gender) and differentiation of research subjects by sex and gender should be considered. Moreover, the SAGER recommendation gives specific advice on how to report gender-sensitive according to the sections of the article (title/abstract, introduction, methods, results, discussion) [45]. Sex and gender analysis might help expose diversity of minorities. Through a qualitative research Asquith et al. identified factors to have transgender and gender diverse (TGD) patients participating in research. Facilitators are: Involving the TGD community and research led by TGD researchers; Barriers are: Research from a “cisgender lens” and that research is not accessible for TGD people [46].

Following, barriers are named explaining reasons for non-implementation of gender sensitivity in research [31]. Reza et al. described several barriers, like women not adequately included in heart failure clinical trials. On patient-level (1) travel burden and absenteeism from work, (2) family and childcare responsibilities, (3) Perception of elevated risk of enrollment and (4) financial, socioeconomic, psychological, cultural and health-related barriers inhibit patients to participate at clinical trials. On trial-level (1) low rates of referral and eligibility screening, (2) lack of sex-related eligibility criteria, (3) homogeneous trial leadership and (4) exclusion of elderly participants are barriers to the enrollment of women in clinical trials [33]. Potential matters for gender-sensitive research being less in the foreground could be depending on the research institution, funder and personal or leader alignment [26]. Researchers may be unaware of the importance of sex and gender considerations, and lack research skills to incorporate them [30]. It would be beneficial if each epidemiologist know the guidelines and recommendations for safeguarding good epidemiological practice (GEP guideline) [47]. The guidelines include recommendations to integrate gender sensitivity (particularly sex and age) for a more structured research process.

Finally, to disseminate gender-sensitive research findings, planning a conference is useful. Safdar & Greenberg described how to organize and conduct conferences with focus on gender sensitivity. The conferences should be divided in three successive steps: (1) Summarizing and consolidating data related to gender-sensitive research to identify gender gaps and to generate a research agenda, (2) consolidating a consensus-driven research agenda that advances gender-sensitive research and (3) building a consortium to disseminate gender-sensitive research results [48].

##### Pharmaceutical sector

In the past, women were long time excluded from research in pharmaceutical and research in general. Basic and clinical research examined cells, tissues and humans were predominantly male. Fertile women as participants in pharmacological studies have been systematically excluded due to the potential risk of abortion or teratogenic side effects [49]. In the 50s and 60s of the last century the thalidomide scandal has changed frame conditions on research and political level in terms of considering sex and gender in research and science. Nevertheless, women are often still underrepresented in first phase trials. In this first phase drug dosages, side effects and safety are determined. In comparison to men, women would generate higher costs in research studies through insurance, management and recruiting a bigger sample to achieve valid study results. Additionally, it is necessary to consider certain attributes of feminine bodies, like more intense hormonal changes, menstrual period, menopause or the intake of contraceptive [20]. Even if a higher proportion of women are included in clinical trials, the results are not reported on a gender-specific basis [49]. Consequently, there are rarely recommendations with specific drug treatment instructions for women and men. Lacking knowledge and evidence of dosage effects on women and men, leads to not considering specific dosages and directions for men and women when prescribing medications [50]. Cassese & Zuber recommended including all the individuals, like man, woman, black, white etc. in drug studies.

The web-based knowledge data base “Janusmed sex and gender” can be utilized to find specific drug dosages for women and men. Janusmed contains non-commercial, evidence-based information on medicines regarding sexual and gender-specific aspects in the treatment of patients. The substances have been systematically reviewed and classified in a standardized way. The classification contains clinically relevant sex-specific differences based on the available evidence. Mainly biological sex differences are assessed [50].

##### Professional association

Professional associations publish and renew guidelines or expert standards for health care professionals, both practitioners and nurses. They consist of expert teams, which typically develop guidelines based on evidence-based literature and expert´s knowledge and opinion to publish guidelines for other health care professionals of the same specialty. The guideline´s content and agenda setting depend on the expert team and on method paper to generate the guideline. Tannenbaum et al. developed a “Structured framework for generating sex-specific guidelines” to facilitate establishment of GSC+ integration in guidelines. The framework is subdivided into five parts: (1) Determine if sex is relevant to guideline development, (2) determine scope, plan, committee membership, (3) assess evidence, (4) develop recommendations and (5) implement guidelines. Existence of sufficient primary research and evidence for the specialty is an essential key factor [51]. Zeitler & Babitsch identified facilitators and barriers to develop gender-sensitive guidelines on political, organizational, and individual/professional level.

Facilitators on the political level are funding of gender-sensitive studies or public financing of guideline development. On the organizational level professional associations providing instructions for inclusion of gender sensitivity and supports prioritization of gender determinants in guidelines. On individual/professional level positive attitude of health care professionals facilitates to consider gender disparities in guideline development.

Barriers on the organizational level are lack of resources and high workload. Impairing factors on the individual/professional level are lack of gender awareness, negative attitude to gender sensitivity and low expectations for additional benefits for patient outcome. Imbalanced group dynamic and increased complexity of guidelines are further inhibiting factors [52].

In guideline-writing panels, women or academic experts of patient-centered care should be involved. Another approach is to have patients participate with their experiences on guideline development [25]. The professional association in the USA developed women-specific guidelines in cardiology. That implies more awareness to GSC+ amongst practitioners and helps implementing those in the daily practice [53]. Guidelines implementation tools are necessary to help end-users (health care professionals and patients) implement recommendations [25].

#### Main dimension – education

In order to implement GSC+, knowledge must be disseminated through education. Measures in different areas of education were identified: Multidisciplinary education, nursing education, transgender education, transgender nursing education, (LGBTQ+) medical education and education in the clinical clerkship.

##### Multidisciplinary education (university and training)

In the dimension of multidisciplinary education, articles were included that contain measures for different professionals with various methodological approaches. Several authors emphasize that it is necessary to integrate GSC+ contents in teaching different healthcare professions, e.g. mandatory integration into the main curricula [26, 54, 55]. Greenberg stated that the absence of representative patient sample in the training experience hinders the ability to achieve precise assessment and preparation for real-world scenarios [56]. According to Siller et al. male students in particular benefit from the integration of GSC+ into the curricula and patient care for all sexes improves through individualized consideration [55]. Furthermore, the importance of interprofessional collaboration in the course of GSC+ was emphasized [54]. Some of the articles contained concrete approaches in education on how GSC+ could be implemented: McGregor et al. mentioned workshops or small group activities for a better understanding of the relevance of sex and gender in theoretical and practical areas [54]. McGregor et al. developed a gender health education tool that contains questions to guide researchers for considering sex and gender health education and research across domains e.g. basic science, pharmacology, epidemiology or public health [54].

Posing inquiries about prejudice could enhance clinical care and equally influence the approach to health issues at a societal level through public health initiatives. Health care profession educates are obliged to uncover these prejudice for their own knowledge and attitude while teaching. It is stated that region-specific, culturally focused education is needed for better healthcare outcomes. Therefore, simulators and enhancing training materials to recognize atypical symptoms in women can be useful, especially related to coronary ischemia [56].

The reviewed articles in the dimension of multidisciplinary education highlight the necessity of integrating sex and gender content into the curricula for various healthcare professions, emphasizing training about cultural differences, interprofessional collaboration, and innovative educational approaches.

##### Medical education and training (university)

The integration of gender-sensitive concepts into medical education and training is essential for preparing future physicians to consider gender-specific differences in diagnosis, treatment, and patient communication.

Various authors have advocated for the adaptation and advancement of medical curricula, emphasizing the essential integration of gender-sensitive content [41, 57–60]. Several specific measures have been developed to implement GSC+ in medical curricula. Clever et al. and Tannenbaum & Moineau described curricular integration and structural anchoring by utilizing top-down and bottom-up processes for the integration of GSC+ at universities [61, 62]. Successful implementation requires a curriculum change team consisting of experts to provide targeted support [63, 64]. In a managerial function, a project manager or change agent should define precise learning objectives, monitor curriculum effectiveness, and promote interdisciplinary discussions on gender knowledge [65, 66].

Teaching GSC+ requires committed and competent teachers with goal-oriented pedagogic structure [67]. Political support and legislation are essential to promote GSC+ in curricula, especially in programs for graduates and future lecturers [68]. The promotion of gender competence and awareness also includes teaching about sexism and gender awareness. Including GSC+ as an integral and mandatory part of the curriculum didactic materials, case studies, reports, international networking, e-learning materials, and web-based knowledge-sharing platforms can be supportive in that regard [58, 69]. Curriculum modification aims to integrate different medical specialties to ensure comprehensive consideration of GSC+ in all areas [41, 70, 71].

Specific concepts for the integration of GSC+ in curricula were proposed such as a 6-step approach for curriculum development [60] and for implementation the 4-Year Sexual Orientation and Gender Identity Curriculum [72], and the “Muchacha Curriculum” [59] as well-proven concepts. Eisenberg et al. proposed a women’s health rotation for final-year medical students to learn about significant sex differences [73]. Curricula should contain instructions on how to teach GSC+ and how to apply the know-how in daily practice [31].

Medical teaching materials also play a significant role in teaching GSC+. Many medical textbooks are still gender-biased, leading to a lack of information about gender differences in disease presentation, diagnosis, and treatment. The existing gender biases in medical textbooks and inadequate integration of women’s health material due to a lack of awareness of gender differences are significant challenges [74, 75]. Therefore, it is recommended that medical curricula boards consider gender-related specialties when selecting books and lecture materials [68]. Overall, attitude changes in society are required instead of focusing on biological knowledge to reduce subconscious prejudice on stereotypes [69]. In addition to mandatory curricular integration, further supportive factors are important, such as training programs for faculty and lecturers, the development of clinical electives, journal clubs, webinars, and online educational programs [52]. E-gender medicine learning modules, communication techniques, and interdisciplinary cooperation are useful components for teaching. Their effectiveness is supported by an interprofessional core team, ongoing education, internal seminars, and universally accessible digital resources [76]. Indirect measures to increase GSC+ include raising awareness, sensitization through gender-sensitive language, and physiological knowledge expanding further than only related to stereotypes [77–79].

Direct measures such as training programs and the development of clinical elective courses are crucial for promoting gender education. Seeland, Templeton and Adreak et al. emphasized the pivotal role of training programs in highlighting gender aspects in medical education [57, 60, 76]. The development of teaching materials and the utilization of digital resources and e-learning platforms are further measures for implementing gender sensitivity in education [59, 78, 79]. Dijkstra et al., Meulen et al., and Henrich et al. pointed out the necessity of these measures for promoting a gender-sensitive curriculum [74, 75, 80]. Raising awareness of gender medicine, promoting consciousness and knowledge acquisition are also considered essential [65, 81, 82]. Indirect measures are e.g. promoting interdisciplinary collaboration, network formation, political support, and funding [61, 70, 83]. Hochleitner, Ton, and Tollemach et al. emphasized the importance of political measures and financial resources to integrate gender medicine into curricula [68, 72, 84]. Direct measures such as training programs and the development of clinical elective courses are crucial for promoting gender education.

Several barriers impede the implementation of GSC+ in medical education and training. A lack of awareness and interest in GSC+ persists [71, 82]. Böckers et al. highlighted the limited interest of decision-makers in implementing gender-sensitive medicine [65]. Clever et al. identified a general lack of awareness, insufficient research funding, and poor interdisciplinary networking [61]. The relevance of GSC+ is often unknown and equated or reduced to the concept of gender equality [81]. Resistance from faculty members, limited teaching time and discussion of incorporating gender sensitivity into the curriculum are further barriers [58, 84–86].

Exploring the barriers to gender education within medical training courses reveals some challenges. Risberg et al. highlighted the risk of ‘knowledge-mediated gender bias’ and emphasized the importance of physician awareness [87]. Böckers et al. noted the limited interest in implementing gender medicine especially among male stakeholders [65]. Despite best-practice examples, Seeland observes inadequate implementation of gender medicine across various regions [88]. Encandela underlined the difficulty of prioritizing gender medicine within curricula due to the scarcity of time and expertise among faculty members [89]. Gaida mentioned concerns regarding absence of formal curricular subjects on gender medicine or sensitivity [90]. The persistence of gender bias in research and practice exacerbates these challenges [78, 79].

##### Nursing education

Measures proposed for the field of medical education can also be applied to nursing education, such as the integration of GSC+ content into nursing curricula and raising students´ and teachers´ awareness of GSC+. In the area of nursing education, various approaches have been proposed to enhance gender-sensitive nursing care. Pai presented a nursing education action program comprising workshops and preparatory activities for educators [91]. Noonan et al. emphasized the importance of developing gender-sensitive nursing curricula [92]. Lindsay & Kolne advocated for integrating gender-sensitive nursing practices into training programs [93], while Klotzbaugh suggested a cultural competency module focused on minorities within advanced practice nursing courses [94].

In the field of nursing education, Lindsay & Kolne identified various barriers hindering the integration of gender-sensitive nursing care [93]. These obstacles encompass a lack of faculty training and experience, challenges originating from perceived or actual gender differences and stereotypes, as well as issues related to binary documentation systems. Additionally, patient preferences for specific gender providers, gender-based discrimination and inappropriate touching leading to uncomfortable situations for nurses are further barriers for nursing professionals. Addressing these barriers necessitates targeted training initiatives and improved documentation protocols to foster respectful and inclusive care environments.

##### Medical education for physicians (postgraduate)

Since most universities do not have gender-sensitive modules implemented in their training programs, it is important that physicians receive postgraduate gender-sensitive education to ensure GSC+. Numerous measures have been proposed to achieve this goal, with various authors recommending measures for postgraduate practitioner education. According to the recommendations of Hsieh et al. postgraduate training programs should incorporate content on women’s health, including monthly lectures, journal clubs, and policy seminars [95]. Additionally, the training of instructors in women’s health is essential to ensure that a thorough understanding of gender-sensitive health aspects is taught [96].

Kling suggested that training programs and lectures should be linked to practical aspects of patient care to highlight the relevance of gender-specific health issues [97]. Furthermore, he emphasized the integration of GSC+ into existing curricula to close the educational gap [54]. The research of Dielissen underlined the importance of interactive teaching methods and suggests tutorials over a 6-months period for graduate medical students to raise awareness of gender differences in health care practice [98, 99].

The use of tools such as the GenderMed-Wiki, a nationwide platform for the exchange of gender-specific knowledge, can be useful to answer upcoming questions of GSC+. The authors also emphasized the need to make gender-sensitive content exam-relevant to increase motivation by engaging with gender-sensitive topics [100].

In addition to the proposed measures, it is important to consider and to address the barriers that may hinder the implementation of GSC+ in postgraduate medical education. Bönte et al. highlighted as a significant barrier that female patients obtain less doctor-patient communication time and are less likely to receive an accurate diagnosis by primary care doctors [101]. This points out a fundamental issue in gender bias within healthcare. Celik identified various barriers at different levels [102]. At the individual professional level, knowledge and skills are lacking for developing routines in GSC+. Additionally, the skepticism towards the importance of GSC+ could impede progress. At the organizational context level, heavy workloads might be a challenging factor for healthcare providers to prioritize GSC+. The timing of implementation can be an influencing factor as integrating new practices into existing systems may require significant time and resources [103].

Nevertheless, addressing these barriers is essential to ensure the successful implementation of GSC+ in postgraduate medical education. Efforts to provide adequate training, raising awareness, and create supportive organizational environments are essential.

Sex- and gender-specific education is crucial in emergency medicine to improve patient outcomes and address personalized healthcare needs. Walter highlighted that sex and gender differences in emergency care are often overlooked in traditional medical training, leading to suboptimal care [104]. There is a need for targeted education that incorporates these differences into core emergency medicine training, recommending practical approaches such as simulation exercises, bedside teaching, and case-based learning to better prepare students and professionals [105]. Willging et al. further underlined that structural barriers and a lack of competency in handling transgender and gender non-conforming patients in emergency settings can lead to delayed or inadequate care, advocating for comprehensive training programs to ensure inclusive, respectful, and effective emergency care for all patients [106].

##### Patient-empowerment

In addition to the education of professionals, patients should also be informed about GSC+ and should be empowered to make GSC+ decisions on their concerns. Previously, a lower percentage of women were involved in clinical studies. Resulting in minor knowledge which led to disadvantages in diagnostics and therapy compared to male participants. Cassese & Zuber recommended that women should be actively participating on their therapy plan and should discuss whether women were included in previous research and therapy development. Based on that more specific prescriptions and therapies could be generated [20]. An information tool, like the HeaRT-App, can support prevention and health promotion of patients, particularly for female patients. The app provides tailored gender-specific recommendations on several health topics, e.g. for vaccinations, cancer screenings, general health, risk factors or physical activities. Greenberg’s study revealed that using the app increases health literacy and reduces the incidence of cardiovascular diseases and the mortality due to cancer and other diseases [107].

##### LGBTQ + education and care

LGBTQ + healthcare should be considered in GSC+. There is still a large evidence gap for the specific needs of transgender people.

Many authors named the curricular integration of topics relating to transgender and LGBTQ + health as a beneficial measure [92, 108–113]. Specific concepts were developed for this purpose, for example Shermann et al. developed a 5-step concept for the integration of transgender healthcare into the curricula [108]. Noonan et al. advocated guidelines for sexual and gender minorities health inclusion in medical schools, also including 7 specific strategies for faculty and administration support, integration of transgender health components into curriculum, and gender-sensitive teaching approaches [92]. Noonan et al. developed the eQuality project, which includes online modules to learn about standardized patient interactions [92]. Coordinated initiatives to integrate LGBTQ + health-related content into all health sciences curricula should be supported to encourage students to develop professional attitudes and behavior concerning care for patients with LGBTQ + backgrounds [114, 115].

Several authors pointed out the need for specific trainings for caregivers and medical students regarding LGBTQ + specific healthcare needs [109, 114, 116–121]. Practice-focused trainings with case study-based learning to transfer theory to clinical practice and competence learning, should be taught [109, 122]. Some authors also mentioned inviting transgender people or other people from the LGBTQ + community to teaching sessions [119, 123]. Learning through standardized protocols and attention to appropriate pedagogical methods was mentioned to ensure high quality in teaching [116]. Scholte et al. recommended the integration from basic biomedical knowledge of transgender people in teaching [118]. Educational games can serve as both teaching activities and assessment tools, providing assessment and instant feedback required in competency-based medical education learning processes [121].

It is important to enhance recognition of transgender patients and their individual health needs. Teachers should be role models for their students. This includes developing gender-sensitive communication skills, such as using the correct terminology and addressing people with their correct pronouns [111, 118, 124]. Henriques et al. created the concept of ASK (Awareness, Sensitivity, Knowledge), which incorporates contents of awareness, sensitivity, and knowledge when learning about a new cultural group, allowing students to identify differences and practice gender-affirming communication techniques [125].

The implementation of transgender and LGBTQ + education and care encounters multiple barriers, influencing both the quality of care and the education of healthcare providers. One major aspect is the lack of training in LGBTQ + health issues of providers, leading to subconscious stigma and discrimination. Health care professionals mentioned to feel occasionally unprepared to address specific needs of transgender and LGBTQ + patients [126]. The professionals further noticed specific sexual minority health issues and incorrect use of terminology [74, 75, 120]. Despite the value of case-based teaching, challenges such as time limitations and the complexity of cases are present. Additionally, changes in teaching culture and insufficient institutional support further complicate the implementation of this teaching method [85, 86, 122]. Health discussions with LGBTQ + patients often focus narrowly on safe sex and sexual transmitted infections, overlooking other important health needs such as mental health and routine screenings.

Curricula often neglect special LGBTQ + health training, so that future physicians do not recognize specific needs of LGBTQ + patients [127]. Particularly, nursing education programs, frequently fail to support gender diversity. This leads to trans invisibility and a lack of recognition of the role gender identity plays in health and well-being [61, 65, 109, 124].

While specific knowledge is lacking in medical school curricula, there is also a need for research on the complex social structure required of clinicians and educators working in gender-affirming medicine [116]. Healthcare professionals often express discomfort with gender-related topics and a need for additional resources [78, 117]. Furthermore, skepticism about the importance of gender in healthcare persists of professionals, creating another barrier to implement gender-sensitive care [79, 118]. Transgender youth with limited parental support are particularly at risk, facing mental health disparities and barriers to accessing gender-affirming care [128].

Addressing these barriers requires comprehensive training, raising awareness, and creating supportive environments within healthcare institutions to ensure the successful implementation of transgender and LGBTQ + education and care. Sequeira et al. suggested that the use of telemedicine for young transgender people can reduce the barrier to get access into healthcare [128].

#### Main dimension – institutions of care and (medical) patient record

GSC+, as part of precision care becomes more important for patient health care. Research findings, political decisions, education, and guidelines for health care professionals support the implementation of GSC+. Explicit translation from theoretical to practical level needs structured strategies and recurring processes. Barriers, such as lack of time, knowledge, training and experience inhibit implementation of inpatient and outpatient care. Clinicians perceive GSC+ as important, even though GSC+ is not a priority in health care practice, yet. Increasing GSC+ in institutions of care and to enhance the staffs´ awareness, following measures can be implemented: Employing a diversity commissioner, implementing working groups addressing diversity or regular training courses and consultations for staff [129].

The German Federal Association of Statutory Health Insurance Physicians published a handbook on how to organize quality circles to enhance GSC+. The aim of implementing the quality circles is to provide impetus and motivation to health care professionals to take greater account of the topic in everyday practice. Initializing, information exchange between health care professionals, various methods, such as case conferences with comparison to guidelines, use of specialist literature as part of a journal club, conducting an expert interview and internet research can be used. Results of the quality circles were summarized. Concluding, agreements are made, which are constantly reviewed by the PDCA (plan, do, check, act)-circle [14].

In context of qualitative interviews with health care professionals in pediatric rehabilitation hospitals Lindsay et al. identified that in their outdated medical documentation system and patient forms only two options, male and female, could be chosen. Particularly for transgender and non-binary patients this could potentially lead to misgendering and upsetting patients. More education for health care professionals is needed in care for transgender and non-binary patients, navigating privacy and ethical concerns and wording (e.g. choice of pronoun) [93]. Transgender becomes more recognized, so it is relevant to educate health care professionals´ awareness.

Therefore, the integration of the two-step method with two options “current identity” and “sex assigned at birth” in (medical) records helps health care professionals to contextualize patients correctly. For medical care it is important to know the medical history of patients, likewise of transgender and non-binary patients. Moreover, preferred name, pronoun preference, and ethnicity should be included as demographic variables in patient records, so that health care professionals can care more gender-sensitively through this knowledge [130]. For implementation changes in daily hospital routine, the implementation process must be accompanied and structured. A change agent can facilitate the change process of implementing new data collection throughout the organization. A structured implementation plan is recommended to be performed within a one-year timeframe and includes technical modifications, staff training and a pilot, expanding and monitoring phase with feedback rounds [131].

On the organizational level, the gender of the health care professionals can play a role regarding to patient’s preference for a certain gender provider, gender-based discrimination, and touching and inappropriate comments [132]. It can be supportive in institutions of care to achieve a balance between female and male health care professionals and to provide GSC+ guidelines for orientation to caregivers on how to handle diversity [21].

Particularly for LGBTQ + people´s access to health care is characterized by stigma and discrimination. Therefore, LGBTQ + specific clinics and providers are relevant institutions of care to ensure access and care for a minority [133].

## Discussion and limitations

### Methodological reflection

GSC+ is part of a patient-centered care regarding more precise and evidence-based medical and nursing care. The implementation of GSC+ is currently insufficient despite well-known implementation measures in the literature, which were identified and analyzed through the present scoping review. The multifariousness and the range of measures of different evidence and dimension levels are shown by the authors with the method of a scoping literature review. Results extending from opinion papers of experts to high-evidence papers and content of politics dimension to content of health care dimension were considered. Overall, the level of evidence ranges between II and V. However, it should be emphasized that systematic literature reviews were explicitly excluded. The authors decided to include exclusively primary literature in the scoping review. The authors’ recommendation is to conduct subsequent systematic reviews per dimension.

Previously, literature research was performed to identify literature reviews on GSC+ to detect the research gap and to define the framework for the planned scoping review. This scoping review connects to the systematic review “Bringing gender sensitivity into healthcare practice: A systematic review” from Celik et al. [13]. Hence, an observation period in databases between 2008 and 2023 was determined for this scoping review. While literature screening a rapid systematic literature review from Khamisy-Farah & Bragazzi about implementation measures and facilitators and barriers in undergraduate, graduate, and post-graduate education was identified [134]. The present review is mainly distinguished by inclusion of LGBTQ + health care contents and considering further dimensions (e.g. politics, research and science, institutions of care) in comparison to the rapid systematic review.

### Content-related reflection

This scoping review demonstrates that the implementation of GSC+ is an interdisciplinary topic that requires a holistic approach. Through systematic search, measures from various derived dimensions were identified, which are closely connected and influence each other. Figure 3 illustrates the identified and derived dimensions in the form of a bull´s-eye diagram.

The outer of the bull´s-eye diagram are the dimensions of policy and research/science, which serve as the framework for subsequent dimensions. The political agenda setting in context to GSC+ has an influence on research by financial support or other incentives. Therefore, it is essential in politics to pursue and promote the topic consistently. For example, since the EU regulation (536/2014 (14)) of April 16, 2014, study participants must be selected to be representative (relating to sex and age) of the population group. Yet, a change in government could change priorities, potentially impede long-term progress in GSC+. Research/science is also fundamental to generate evidence that is needed to transfer GSC+ in health care practice. A useful measure to ensure the consideration of GSC+ in research could be the mandatory inclusion of gender specific differences in studies. Additionally, obligatory training on GSC+ topics for researchers and instructions for funding applications to consider gender could be beneficial. Examples from Austria and Canada show that online modules in various research areas support researchers in considering GSC+.

In the pharmaceutical sector, for instance, it is important to state differences in the effectiveness of drugs between women and men. Generated evidence of GSC+ must be translated into actionable recommendations within medical guidelines and nursing expert standards. Furthermore, guidelines and nursing expert standards should contain concrete course of action for practice.

Education is another essential dimension, acting as a catalyst to provide existing knowledge to future generations of healthcare professionals. The compulsory inclusion of GSC+ content in the core curricula of all universities should be considered [41, 57–60]. Implementing gender-sensitive content would ensure an equal level of education for all students in this regard. Currently, GSC+ modules in medical school are often only offered as elective subjects and therefore not attended by all students.

The center of the bull´s-eye diagram is the implementation of GSC+ in healthcare. Various concepts for this were proposed. Some authors support a top-down process from organizations or leaders like chief physicians to implement GSC+. Others advocate for a bottom-up approach. Concrete concepts for implementation were also suggested, such as employing a change agent. In conclusion, implementation should be supported by the entire organization (e.g. hospitals).

It applies to all dimensions that the topic of GSC+ is lacking awareness. This is particularly important in the context of addressing LGBTQ + healthcare needs.

### Reflection of transfer and implications

The question is how to bring gender sensitivity into the different dimensions. In Germany, several initiatives and individuals facilitate and support GSC+. A holistic approach overall dimensions is not pursued for years. In the HeartGap study, it is perceived that one dimension waits until the other begins to consider gender sensitivity and vice versa. In summary, the dimensions build on each other and the foundations are provided by policy and research. An implementation will not work through intrinsic motivation alone although increasing awareness is still important.

The current care situation is characterized by stress, time pressure, overtime, a shortage of skilled staff, and sick leave. Patient routines are performed; it is usually not possible to care for patients individually. Influencing factors are the reimbursement system in the outpatient and inpatient sector, the unattractiveness of the nursing profession, or the mismanagement of the patients in the system. Modifying the influencing factors or enacting laws relating to GSC+ would be an important implementation step. A strategy developed in a participatory process with stakeholders target-oriented from outer to inner setting (Fig. 3) would be recommended.

The focus of the strategy building should be on enhancing the quality of patient care through implementing GSC+. Nursing and medical activities should be performed following the evidence-based guidelines and expert standards. These aim to support healthcare professionals in the effective and efficient performance of their activities. According to § 113a SGB XI, nursing expert standards is binding for all care homes and care services in Germany. Medical guidelines are not legally binding. Physicians can deviate from the treatment recommended in the guideline if they decide that it is not suitable for a particular patient. However, any deviations should be justified. Overall, there is no monitoring whether guidelines are implemented by healthcare professionals, yet. Furthermore, there are quality indicators that are used as measuring instruments in the outpatient and inpatient sectors, but which do not initiate quality competition in healthcare sector to increase quality. Transparent competition for patients could lead healthcare professionals and institutions of care to implement GSC+ more widely. This requires more GSC+ content to be included in the guidelines and nursing expert standards, and quality indicators and their consistent implementation and consideration. For example, obtaining a gender certificate creates transparency for patients and educates healthcare providers to implement GSC+.

In addition to the gender health gap, there are also gaps in other areas, such as the gender pay gap, gender time gap, gender pension gap, gender care gap, or gender data gap. Although men can also be disadvantaged, women are more frequently affected. It is a task for the whole of society to identify and eliminate injustices and inequalities. The current social discussions about minorities and disadvantages can be a catalyst for breaking gender health gaps.

## Conclusion and future directions

The implementation of GSC+ is a cross-dimensional task. It is a crucial step towards equitable and evidence-based healthcare. Significant measures have been identified across the dimensions of politics, research, education, and healthcare institutions, but their adoption remains fragmented and inconsistent. This highlights an urgent need for a comprehensive and binding strategy to ensure GSC+ in healthcare systems.

Policy frameworks must go beyond temporary initiatives and demonstrate sustained commitment by mandatory requirements, targeted funding, and clear accountability mechanisms. Successful example, such as gender-specific public-funded research institutes e.g. in Canada, underline the impact of political will and structured approaches. These could be models for adoption in Germany. In research, the lack of mandatory gender analysis leads to gaps in knowledge and hinders progress in practice. Research funders and institutions must enforce the inclusion of gender considerations as standard criteria in all projects. Researchers´ guidelines, such as the SAGER framework, and tools to facilitate gender-specific literature searches must be systematically applied. Interdisciplinary research teams with balanced gender representation can further improve the diversity and depth of scientific output. In education, integrating GSC+ into medical and nursing curricula remains insufficient. Gender-sensitive content in training must transition from elective to compulsory status, ensuring that all healthcare students obtain the necessary competencies to address diverse patient needs. Institutions must provide educators with tools and training to teach GSC+ effectively to facilitate interprofessional collaboration and cultural competence. At the institutional level, healthcare providers must adopt proactive measures, such as employing equal opportunity officers, updating patient record systems, and facilitating a culture of inclusivity. In addition to structural changes in institutions of care, cultural transformation is needed. The establishment of quality circles and the integration of GSC+ into routine workflows represent practical steps.

Ultimately, the persistent lack of awareness and systemic resistance to GSC+ must be actively confronted. A unified, cross-dimensional approach, driven by strong leadership and enforced through transparent legislation, is essential to achieve progress in implementing GSC+. Fragmented solutions characterize the status quo in implementing GSC+. Implementation measures can be divided into incentives and compulsions. Incentives are e.g. funding, reimbursement, or further education. Compulsions are e.g. compulsory inclusion of GSC+ content in the core curricula of all universities, part of guidelines and expert standards, or mandatory gender balance among research or health care professionals teams. A GSC+ strategy should consist of a combination of incentives, but also compulsions to ensure GSC+ in practice. With concerted efforts, GSC+ can become a cornerstone of patient-centered care, leading to better health outcomes for all and a more equitable healthcare future.

## Supplementary Information

Supplementary Material 1.

## Data Availability

No datasets were generated or analysed during the current study.

## Abbreviations

EBM: Evidence–based medicine
EBN: Evidence–based Nursing
GEP guideline: Guidelines and recommendations for safeguarding good epidemiological practice
GSC+: Gender–sensitive Care
LGBTQ+: lesbian, gay, bisexual, transgender and queer + people
PDCA circle: plan, do, check, act–circle
TGD: transgender and gender diverse
