# Supplementary material for: Multifarious approaches of implementation to transfer gender sensitivity in health care practice: a scoping review
Source: BMC Health Serv Res. 2025 Jun 28;25:825. doi: 10.1186/s12913-025-13032-w (PMC12206366; doi:10.1186/s12913-025-13032-w)
Supplement: Supplementary file 1 — Supplementary Material 1. [file 12913_2025_13032_MOESM1_ESM.docx]

Appendix. Overview of results

| **Author(s) / year** | **Type of participants** | **Sample size /**  **study design** | **Intervention/**  **measure** | **Outcome** | **Intervention effect** | **Main and sub-dimension** | **3 main facilitators** | **3 main barriers** |
| --- | --- | --- | --- | --- | --- | --- | --- | --- |
| [14] Oertelt-Prigione et al. 2017 | Expert stakeholders | 23 experts in discussion rounds (workshops) | Workshops | Identifying overarching goals, the most urgent needs of and stakeholder for gender-sensitive public health; general communication and promotion strategies | Identified the first systematic assembly of gender-sensitive implementation strategies in public health | **Politics** | 1. Structured consensus-building process involving interdisciplinary stakeholders  2. Development of actionable frameworks and tools for policy and practice  3. Potential for political uptake through clearly communicated implementation strategies | 1. Lack of existing standardized methodologies for gender-sensitive implementation  2. Low political awareness of the relevance of sex/gender in public health prior to the initiative  3. Uncertainty regarding structural anchoring and long-term sustainability of outcomes |
| [15] González Vélez et al. 2020 | Expert informants from Peru, Guatemala, Guyana | 37 informants, semi-structured qualitative interviews | 7 categories considered essential to advance gender mainstreaming in the health sector | Successful gender mainstreaming requires a more strategic and transformational agenda | highlights structural conditions and lessons for sustainable gender mainstreaming | **Politics** | 1. Existence of legal and policy frameworks for gender mainstreaming in health  2. Willingness of some institutional actors to support gender-related reforms  3. Engagement of health professionals with awareness of gender equity goals | 1. Persistent societal power imbalances and discrimination limiting systemic change  2. Political instability and lack of institutionalized financial support  3. Fragmentation and complexity within health systems hindering implementation |
| [16] Gagliardi et al. 2020 | Not applicable (policy content analysis) | 30 canadian policy documents, qualitative content analysis of government policies to assess if and how they addressed patient-centered care for women (PCCW) | Equipping policy-makers or healthcare professionals with the tools to develop PCCW-informed policies or to adapt policies into PCCW-tailored healthcare services | PCCW has not been prioritised by policy-makers.These identified gaps represent opportunities by which government policies could be developed or strengthened to support PCCW. | Identified policy-level gaps and opportunities for PCCW advancement | **Politics** | 1. Existing frameworks for PCC (e.g. McCormack et al.)  2. Some policies acknowledged social determinants of women’s health  3. Recognition of collaborative and culturally sensitive care models | 1. Lack of concrete implementation guidance for PCCW  2. Minimal differentiation between men’s and women’s health needs  3. Absence of content on key issues such as violence, stigma, and discrimination |
| [17] Mazure & Jones 2015 | Not applicable (policy and literature analysis) | Review of 20 years of U.S. policies on inclusion of women and sex/gender analysis in biomedical research | Identified ongoing underrepresentation of women and lack of sex/gender analysis in studies | Not nearly enough progress has been made to intergrate women and men in clical trials | Demonstrates limited impact of 20 years of policy efforts and highlights need for stronger implementation mechanisms | **Politics** | 1. Legislative mandates (e.g., NIH Revitalization Act)  2. Establishment of NIH Office of Research on Women's Health  3. Development of specialized research centers | 1. Slow policy implementation  2. Cultural resistance in scientific community  3. Lack of mandatory sex/gender analysis in studies |
| [18] Weber 2008 | Not applicable | Not applicable  (policy and administrative analysis) | Implementation of gender mainstreaming in health policy in North Rhine-Westphalia (Germany) | Identified structural preconditions, developments, and factors for successful implementation | Emphasized need for structural anchoring and cross-sectoral collaboration | **Politics** | 1. Political commitment 2. Institutionalization of gender mainstreaming 3. Cross-sectoral cooperation | 1. Limited resources 2. Lack of gender awareness 3. Resistance in established structures |
| [19] Cassese & Zuber 2011 | Not applicable | Not applicable (policy and literature analysis | Review of gender representation in clinical trials and regulatory frameworks | Identified underrepresentation of women in clinical trials and lack of sex/gender analysis | Highlights policy and regulatory gaps and calls for structural change to support sex/gender inclusion in clinical trials | **Politics** /  **Education** (Patient-empowerment) | 1. EU and WHO initiatives promoting gender inclusion  2. Recognition of sex/gender differences in health outcomes  3. Development of methodological standards and reporting requirements for sex/gender integration in clinical trials | 1. Historical male dominance in clinical research  2. Lack of mandatory sex/gender data analysis  3. Institutional inertia and resistance to change |
| [20] Binder-Fritz & Rieder 2014 | Not applicable | Not applicable (conceptual analysis) | Analysis of the interplay between gender, socioeconomic status, and ethnicity in health and migration contexts | Highlighted the need for intersectional approaches in healthcare to address overlapping social determinants | Emphasized the importance of considering multiple social factors in healthcare provision | **Politics** | 1. Recognition of intersectionality  2. Awareness of diverse patient backgrounds  3. Emphasis on culturally and diversity sensitive care | 1. Lack of integrated policies  2. Insufficient training on diversity  3. Systemic barriers in healthcare access |
| [21] Goldenberg et al. 2020 | Transgender and gender-diverse individuals across the U.S. | ~28,000 participants; cross-sectional survey (2015 U.S. Trans Survey) | Analysis of state-level transgender-specific policies and their association with healthcare avoidance due to fear of mistreatment | Living in states with more protective policies was associated with reduced odds of avoiding healthcare | Protective policies linked to increased healthcare access; restrictive policies associated with higher avoidance | **Politics** | 1. Protective state policies  2. Inclusion of gender identity in nondiscrimination laws  3. Transgender-inclusive Medicaid coverage | 1. Restrictive policies (e.g., broad religious exemptions)  2. Exclusion of transgender care in Medicaid  3. Fear of mistreatment in healthcare settings |
| [24] Gagliardi et al. 2019 | Not applicable (guideline content analysis) | 27 clinical guidelines (18 depression, 9 cardiac rehabilitation); content analysis | Assessment of inclusion of patient-centred care (PCC) elements and women's health considerations in clinical guideline | All guidelines mentioned at least one PCC domain; 14 (51.9%) mentioned women's health, primarily referencing social determinants without actionable guidance | Identified gaps in comprehensive PCC and women's health content; emphasized need for improved guideline development practices | **Politics**  (Career advancement) | 1. Existing PCC frameworks (e.g., McCormack et al.) 2. Awareness of gender-related social determinants 3. Recognition of need for better guideline development | 1. Limited stakeholder involvement 2. Lack of specific PCC recommendations for women 3. No use of a women’s health-specific framework |
| [25] Hentzen et al. 2022 | Universities, educational institutions, national & EU education policies | Mapping study (qualitative/narrative overview) | Integration of sex and gender aspects in medical and scientific education | Overview of current status of sex/gender integration in curricula; recommendations for improvement | Suggested systematic integration of sex/gender into medical and life science education | **Politics**  (Career advancement)  **Research/Science**  (GSC+ in research process)  **Multidisciplinary education** (university and training) | 1.Gender balance among researchers  2.Transnational collaborations on the integration  3.Mixed research team promote awareness | 1. Lack of systematic integration into core curricula 2. Efforts on individual motivation 3. Absence of unified national strategies |
| [26] Carnes et al. 2017 | Not applicable (analysis of endowed chair positions and perceptions) | 19 endowed chairs identified; 15 incumbents responded to survey | Examination of the role of endowed chairs in women's health to advance women's leadership and health initiatives | Increase in endowed chairs from 7 (2013) to 19 (2015); incumbents perceived chairs as enhancing leadership and institutional focus on women's health | Endowed chairs associated with increased women's leadership and promotion of women's health research and education | **Politics**  (Career advancement) | 1.Institutional commitment to women's health (helping advance women into leadership positions)  2. Recognition of endowed chairs' role in promoting leadership  3. Positive perceptions of incumbents regarding impact on women's health initiatives | 1. Not all incumbents felt chairs enhanced their leadership  2. Lack of fundraising expertise among some leaders  3. Potential conflation of gender and status affecting outcomes |
| [27] Oldhafer 2019 | Not applicable | Not applicable  (conceptual analysis) | Discussion on integrating gender medicine into clinical practice through change management strategies | Emphasized the need for structured change management to implement gender-sensitive approaches in medicine | Proposed that successful integration requires awareness, leadership commitment, and organizational support | **Politics**  (Career advancement) | 1. Women in leader positions lead to positive effects for GSC+  2. Structured change management processes  3. Interdisciplinary collaboration | 1. Resistance to change among staff  2. Lack of awareness about gender-specific issues  3. Insufficient training and resources |
| [28] Gansefort & Jahn 2016 | Epidemiologists in Germany | 276 participants; online survey | Survey on experiences, attitudes, and needs regarding gender-sensitive research | 70% had experience with gender-sensitive research; 83% expressed interest in future engagement | Identified need for training and support to enhance gender sensitivity in epidemiological research | **Research/Science**  (GSC+ in research process) | 1. Existing experience among researchers  2. Interest in further engagement  3. Recognition of importance of gender aspects in research | 1. Lack of training opportunities  2. Limited integration of gender aspects in research processes  3. Insufficient institutional support |
| [29] van Hagen et al. 2021 | Not applicable (analysis of research proposals) | 38 research proposals; qualitative content analysis | Evaluation of sex and gender considerations in public health research proposals using CIHR criteria | Found that both sex and gender were poorly considered; mentions were often inconsistent and fragmented | Highlighted the need for improved integration of sex and gender in research proposals | **Research/Science**  (GSC+ in research process) | 1. Gender mainstreaming at all levels of research policy-making  2. Awareness of the importance of sex and gender considerations  3. Journals should aim to publish gender-sensitive research | 1. Poor consideration of sex and gender in proposals  2. Inconsistent and fragmented mentions  3. Lack of structured guidance for researchers |
| [30] Regensteiner et al. 2020 | Not applicable | Not applicable  (conceptual analysis) | Discussion on the importance of integrating sex as a biological variable (SABV) into biomedical curricula | Highlighted the need for curriculum development to include SABV to improve research quality and health outcomes | Emphasized that incorporating SABV into education can lead to more rigorous and reproducible science | **Research/Science**  (GSC+ in research process) | 1. Recognition of SABV's importance in research  2. Support from institutions and funding agencies  3. Availability of existing frameworks for curriculum development | 1. Lack of standardized curricula addressing SABV  2. Limited faculty training on SABV topics  3. Resistance to curricular changes within institutions |
| [31] Sapir-Pichhadze & Oertelt-Prigione 2023 | Not applicable | Not applicable  (conceptual model) | Development of the P32 model: a sex- and gender-sensitive framework for evidence-based precision medicine in kidney transplantation | Identified gaps in addressing sex/gender in transplantation research and care; proposed an integrative model for improvement | The P32 model offers a structured approach to integrating sex/gender in research, clinical practice, and policy | **Research/Science**  (GSC+ in research process) | 1. Recognition of sex/gender impact on transplant outcomes  2. Sex/gender in every single step from research question to data analysis to clinical care  3. Emphasis on interdisciplinary collaboration | 1. Lack of sex-disaggregated data  2. Limited integration into clinical guidelines  3. Resistance to change in clinical routines |
| [32] Reza et al. 2022 | Not applicable | Not applicable  (narrative review) | Analysis of sex- and gender-specific barriers to women's enrollment in heart failure clinical trials and proposed strategies to enhance inclusion | Identified persistent underrepresentation of women in heart failure trials; outlined patient- and trial-level barriers; proposed interventions across the clinical trial lifecycle | Emphasized the need for targeted strategies to improve women's participation in heart failure clinical research | **Research/Science**  (GSC+ in research process) | 1.Implementation of sex- and gender-specific enrollment strategies  2. Inclusion of women in trial leadership roles  3. Development of tailored educational and consent materials for women | 1.Historical exclusion of women from early-phase trials  2. Lack of awareness and access to clinical trials among women  3. Socioeconomic and logistical challenges limiting participation |
| [33] Schiebinger & Klinge 2018 | Not applicable | Not applicable  (conceptual analysis) | Development of methods for sex and gender analysis in health and medicine; presentation of case studies demonstrating the impact of such analyses | Integration of sex and gender analysis enhances research quality and leads to new discoveries in biomedical and health research | Demonstrated that considering sex and gender leads to improved outcomes and innovations in health research | **Research/Science**  (GSC+ in research process) | 1. Development of state-of-the-art methods for sex and gender analysis  2. International collaboration among experts from various disciplines  3. Support from major institutions like the European Commission and NIH | 1. Historical lack of sex and gender considerations in research  2. Limited integration of sex and gender analysis into existing research frameworks  3. Need for training researchers in applying sex and gender analysis methods |
| [34] McGregor & Choo 2012 | Not applicable | Not applicable  (conceptual analysis) | Discussion on the historical neglect and future opportunities of gender-specific medicine in emergency care | Highlighted the importance of integrating sex and gender differences into medical research and practice to improve patient outcomes | Emphasized that considering sex and gender leads to more accurate diagnoses and effective treatments | **Research/Science**  (GSC+ in research process) | 1. Federal funding  2. Increasing advocacy for inclusion of gender-specific data in research  3. Development of educational programs focusing on gender-specific medicine and institutes for women´s health (sex- and gender-specific research library) | 1. Historical lack of awareness and consideration of gender differences in medical research  2. Limited incorporation of gender-specific content in medical curricula  3. Resistance to change in established clinical practices |
| [35] Oertelt-Prigione 2020 | Not applicable | Not applicable (conceptual commentary) | Analysis of the state of sex- and gender-sensitive medicine (SGSM) and call for systematic integration of gender dimensions in research and healthcare | Highlights the lack of tools for gender analysis, clarifies differences between sex and gender, and emphasizes structural influences on health outcomes | Proposes conceptual and ethical imperatives to advance SGSM | **Research/Science**  (GSC+ in research process) | 1. Growing support from funders and journals  2. Increased clarity on SGSM principles  3. Integration of social science concepts into medical discourse | 1. Ongoing confusion between sex and gender in practice  2. Lack of systematic gender analysis tools  3. Risk of reinforcing stereotypes when analyzing gender |
| [36] Raparelli et al. 2022 | Internal medicine researchers and SIMI working group members | Consensus development via WG meetings, congress input, literature review | Development of the “SIMI Gender 5 Ws Rule” as a conceptual and operational framework for SG-integration in clinical IM studies | Provided a structured guide (Who, What, Where, When, Why) and defined a gender core dataset to support sex/gender-based research | Framework enables improved SG integration in IM research design and data collection | **Research/Science**  (GSC+ in research process) | 1. Multistake-holder consensus and literature-based framework  2. Operationaliza-tion of gender variables into a core dataset  3. Alignment with international efforts (e.g., GENESIS-PRAXY, GOING-FWD) | 1. Lack of standardized gender measures across studies  2. Practical barriers to collecting complex gender variables  3. Limited awareness or training among clinical researchers |
| [37] Eifert et al. 2013 | Not applicable | Not applicable (conceptual/methodological overview) | Discussion of gender lens tools and sex/gender integration in cardiovascular research and training | Advocates for structured SGBA in research; introduces practical tools (e.g. gender lens, SGBA checklist) and argues for their application | Aims to raise awareness and support structural change in research practice | **Research/Science**  (GSC+ in research process) | 1. Introduction of SGBA methodology and tools  2. Institutional support (e.g. ESC guidelines)  3. Framing gender as integral to innovation and quality in science | 1. Low use of SGBA in current cardiovascular research  2. Confusion between sex and gender terminology  3. Structural and ethical challenges in including pregnant women in trials |
| [38] Song et al. 2016 | Not applicable (tool development and validation via literature samples) | Validation against 189 manually selected articles | Development and validation of a search tool for SGSH (sex and gender specific health) literature in PubMed | Tool showed higher sensitivity/specificity compared to basic keyword searches; reduced effort to find SGSH-relevant literature | SGSH search tool retrieved 53–65% of reference articles; significantly outperformed standard keyword searches | **Research/Science**  (GSC+ in research process) | 1. Open-access, user-friendly tool based on PubMed  2. Structured, reproducible search strategy using MeSH/text terms  3. Validated against curated reference bases (diabetes/stroke) | 1. Lack of standardized MeSH terms for sex/gender differences  2. Difficulty retrieving relevant articles due to vague terminology  3. No automatic indexing for SGSH content in PubMed |
| [39] Oertelt-Prigione et al. 2014 | Not applicable | Tool development and systematized content analysis | Creation of GenderMedDB – an interactive, searchable database for sex/gender-specific medical literature | Indexed >11,000 publications with SGSM content; offers search tools, statistical summaries, and community functions | Improved access to SGSM literature; fosters knowledge sharing and research; usage and outcome data not yet formally evaluated | **Research/Science**  (GSC+ in research process) | 1. Broad scope and open access functionality  2. Systematic indexing and expert validation  3. User participation through uploads and forums | 1. Ambiguity in sex/gender terminology complicates classification  2. Underrepresen-tation of some fields (e.g. surgery)  3. No automated quality rating of included studies |
| [40] Miller et al. 2013 | Medical educators, curriculum developers, institutional leaders | Workshop with representatives from 13 U.S. medical and public health schools | 2-day workshop to integrate sex and gender concepts into medical education | Identified curricular gaps; developed strategies and recommendations for integration | Raised awareness; proposed actionable steps for curriculum enhancement | **Research/Science**  (GSC+ in research process)  **Education**  (Medical education and training (university)) | 1. Institutional support 2. Interprofessional collaboration 3. Availability of existing resources | 1. Curriculum overload  2. Lack of faculty training  3. Limited awareness of sex and gender importance |
| [41] Miller & Bahn 2013 | Mentors and scholars in interdisciplinary sex/gender research (BIRCWH program) | Case study; program description (no sample size specified) | Implementation of an interdisciplinary mentoring program (BIRCWH) to support sex/gender-based research | Describes structural components, mentoring strategies, and lessons learned in team development and evaluation | Improved mentoring effectiveness, scholar satisfaction, and interdisciplinary collaboration; long-term outcome data not reported | **Research/Science**  (GSC+ in research process) | 1. Structured interdisciplinary mentoring model  2. Institutional integration via CTSA collaboration  3. Tailored evaluation and feedback processes | 1. Lack of mentors with sex/gender expertise  2. Institutional silos hindering interdisciplinary work  3. Time/resource constraints for clinical mentors |
| [42] Pöge et al. 2019 | Stakeholders in health reporting and public health research | Participatory project; conceptual and methodological framework | Development of a participatory, intersectional framework to support gender-sensitive public health research and reporting in Germany | Identified structural and methodological requirements for sustainable gender-sensitive and intersectional health reporting | Framework intended to guide future research and reporting standards | **Research/Science**  (GSC+ in research process) | 1. Participatory approach involving multiple stakeholders  2. Methodological integration of intersectionality  3. Institutional awareness and cooperation | 1. Lack of disaggregated and intersectional data  2. Limited transferability of methods across institutions  3. Challenges in implementation within existing structures |
| [43] Starker et al. 2016 | Not applicable (population-level health reporting) | Not applicable  (policy reflection and synthesis of national health report) | Assessment of challenges and needs in sex/gender-sensitive health reporting based on the national men’s health report | Identified structural gaps in gender-sensitive data availability and interpretation; called for sustainable, equity-driven reporting systems | Offered strategic recommendations for improving gender-sensitive public health reporting | **Research/Science**  (GSC+ in research process) | 1. Political momentum through men’s health reporting  2. Institutional recognition of gender relevance in reporting  3. Intersectoral approach to public health equity | 1. Lack of gender-disaggregated routine data  2. Limited awareness in reporting institutions  3. Underrepresentation of male-specific health topics in traditional frameworks |
| [44] de Castro et al. 2016 | Not applicable (commentary by guideline authors) | Not applicable (policy and editorial framework discussion) | Introduction and rationale of the SAGER reporting guidelines for sex/gender equity in research publication | Guidelines provide structured recommendations for reporting sex/gender in research; intended to improve transparency and applicability | Editorial uptake anticipated; actual guideline implementation impact not assessed in this paper | **Research/Science**  (GSC+ in research process) | 1. Clear editorial framework for sex/gender reporting  2. Support by international editor associations (EASE)  3. Practical applicability across disciplines and study types | 1. Lack of awareness among researchers/editors  2. Confusion between sex and gender terminology  3. Underrepresentation of sex/gender aspects in editorial policies |
| [45] Asquith et al. 2021 | Transgender and gender diverse (TGD) primary care patients | 28 participants in 4 focus groups; qualitative design | Exploration of barriers and facilitators to clinical research participation for TGD patients to inform a longitudinal cohort study | Identified unique motivators (e.g. community connection, TGD-led research) and barriers (e.g. distrust, "cis lens", mislabeling) | Results directly informed study design and participatory methods for future research | **Research/Science**  (GSC+ in research process) | 1. TGD-led research teams and community involvement  2. Integration of research into routine care  3. Emphasis on helping the TGD community | 1. Research perceived as exploitative or “cisgender-framed”  2. Mistrust regarding data use and privacy  3. Feeling excluded or "not trans enough" for inclusion |
| [47] Safdar & Greenberg 2014 | Emergency care researchers, clinicians, stakeholders (incl. federal agencies) | 133 participants; structured consensus conference | Development of a national research agenda for gender-specific emergency care via a multi-step consensus process | Identified 7 priority domains; produced a structured research agenda and built interdisciplinary networks | Enabled new research collaborations; improved agenda-setting; long-term research impact intended but not yet measured | **Research/Science**  (GSC+ in research process) | 1. Rigorous consensus methodology (nominal group technique)  2. Multidisciplinary stakeholder involvement  3. Federal and institutional support | 1. Lack of sex-disaggregated emergency data  2. Underrepresentation of gender variables in current research  3. Editorial and publication challenges for gender topics |
| [48] Holge-Hazelton & Malterud 2009 | Not applicable | Not applicable (commentary / policy and practice reflection) | Argumentative analysis of how gender perspectives can and should be integrated into medical knowledge, education, and practice | Demonstrates how implicit gender norms influence clinical reasoning and practice; advocates for epistemological openness to gender | Calls for critical awareness and inclusion of gender-sensitive epistemology | **Research/Science**  (Pharmaceutical sector) | 1. Reflexivity in clinical practice  2. Recognition of gendered power structures in healthcare  3. Commitment to epistemological diversity in medicine | 1. Biomedical bias against gender as a “soft” factor  2. Structural resistance to interdisciplinary integration  3. Lack of institutional commitment to gender perspectives |
| [49] Karlsson Lind et al. 2017 | Not applicable (no individual-level participants) | Descriptive database and tool development (Janusmed Sex and Gender) | Development of an evidence-based knowledge base for sex- and gender-aware drug prescribing in Sweden | Created a structured, widely accessible tool covering ~300 substances; used for clinical decision support and education | Facilitated gender-sensitive prescribing; usage and satisfaction high, though clinical impact not formally evaluated | **Research/Science**  (Pharmaceutical sector) | 1. Governmental and institutional support (SALAR, Stockholm Council)  2. Integration into regional prescribing tools  3. Structured, evidence-based update process | 1. Lack of sex-disaggregated data in drug trials  2. Limited awareness among healthcare professionals  3. Complexity in distinguishing sex vs. gender aspects |
| [50] Tannenbaum et al. 2019 | Not applicable | Not applicable (conceptual framework and review) | Development of a structured framework to integrate sex-specific evidence into clinical practice guidelines | Clear roadmap for guideline developers on how to assess, report, and implement sex-specific evidence; identification of implementation gaps | Proposed framework used by Canadian societies in pilot guideline development | **Research/Science**  (Professional association) | 1. Structured step-by-step guideline integration model  2. Rising availability of sex-disaggregated trial data  3. Institutional buy-in (e.g. CCS pilot project) | 1. Underrepresen-tation of women in trials  2. Lack of sex-disaggregated outcomes in studies  3. Confusion between sex and gender in guideline processes |
| [51] Zeitler & Babitsch 2018 | Experts involved in developing or reviewing clinical practice guidelines in Germany | 10 expert interviews; qualitative study | Exploration of barriers and facilitators for integrating sex/gender aspects into clinical guidelines | Revealed key structural and attitudinal factors influencing gender sensitivity in guideline development | Results informed strategies for improved integration in future guideline processes | **Research/Science**  (Professional association) | 1. Awareness of gender relevance among some stakeholders  2. Existing models for integrating sex/gender in certain disciplines  3. Motivation for quality improvement | 1. Lack of data and evidence in clinical studies  2. Perception of gender as non-relevant  3. Absence of methodological standards or mandates |
| [52] Hochleitner et al. 2013 | Medical students and faculty at the Medical University of Innsbruck | Descriptive case study (no fixed sample size reported) | Integration of gender medicine into the mandatory medical curriculum via a dedicated module and cross-curricular content | Gender medicine became a visible and structurally anchored part of the curriculum; faculty and students increasingly engaged with the topic | Long-term integration achieved; cultural change fostered; increased awareness and sustainability reported | **Education**  (Multidisciplinary education (university and training)) | 1. Institutional leadership and top-down support  2. Gender medicine embedded in exam-relevant modules  3. Faculty sensitization and training | 1. Perception of gender medicine as ideology or women’s issue  2. Initial resistance from faculty  3. Lack of established teaching materials |
| [53] McGregor et al. 2019 | Health professionals, educators, and students across five disciplines | 246 participants; national summit with pre-/post-surveys | SGHE Summit: Workshops, lectures, resources on sex/gender in curricula | Increased awareness, knowledge, and commitment to integrate SGHE | Improved attitudes and intentions to include SGHE in education | **Education**  (Multidisciplinary education (university and training)) | 1. High participant motivation 2. Interprofessional collaboration 3. Availability of resources/toolkits | 1. Low existing curricular integration 2. Institutional inertia 3. Dependence on individual efforts |
| [54] Siller et al. 2018 | Medical and allied health students (clinical and lab-focused) | 483 students; cross-sectional survey | Attendance at Gender Medicine lectures | Increased gender awareness, especially in lab-focused students and male students | Positive association between lecture attendance and gender sensitivity | **Education**  (Multidisciplinary education (university and training)) | 1. Tailored and compulsory lectures  2. Receptive student groups  3. Institutional support | 1. Limited curriculum integration 2. Gender biases 3. Resource constraints |
| [55] Greenberg & Pierog 2009 | Not applicable (analysis of educational materials) | Evaluation of AHA ACLS materials (2006 edition) | Assessment of race and gender representation in ACLS training resources | Identified underrepresentation of women and minorities in training materials | Highlighted need for inclusive representation to improve cultural competence | **Education**  (Multidisciplinary education (university and training)) | 1. Established ACLS training framework  2. Recognized need for cultural competence  3. Potential for material revision | 1. Predominant portrayal of white male patients 2. Lack of diversity in mannequins and case scenarios 3. Missed opportunities to depict diverse populations |
| [56] Templeton 2013 | Not applicable | Not applicable  (commentary/editorial) | Advocacy for integrating women's health and sex/gender medicine into medical education | Highlighted gaps in curricula; called for increased inclusion of sex/gender topics | Raised awareness among educators and institutions | **Education**  (Multidisciplinary education (university and training)) | 1. Growing recognition of sex/gender differences 2. Support from professional organizations 3. Develop curricula | 1. Lack of formal integration of sex and gender content into medical curricula  2. Insufficient availability of standardized teaching materials and clinical examples  3. Low institutional priority and resistance to curricular reform |
| [57] Yang 2020 | Medical and nursing educators and practitioners | 50 participants; cross-sectional survey | Assessment of essential vs. frequently taught gender concepts in curricula | Identified gaps between essential gender topics and those frequently taught | Highlighted need for curriculum reform to include critical gender concepts | **Education**  (Multidisciplinary education (university and training)) | 1. Educator awareness and gender competency  2. Recognition of structural issues  3. Emphasis on gender equity | 1. Time constraints  2. Curriculum overload  3. Lack of standardized content |
| [58] Reza et al. 2020 | Cardiovascular fellows-in-training (FITs) and faculty | Descriptive implementation report (pooled evaluation: n=34) | Implementation of the MUCHACHA curriculum – a women’s CVH-focused educational program in a cardiology fellowship (lectures, case conferences, clinic rotations) | Increased exposure to sex- and gender-specific CVH topics; high FIT satisfaction; formal integration into curriculum | 97% of FITs rated didactics as very good/excellent; program expanded knowledge and fostered collaboration; long-term clinical impact not measured | **Education**  (Multidisciplinary education (university and training)) | 1. FIT-led initiative with strong faculty support  2. Integration of case-based, multidisciplinary learning  3. Alignment with national training goals and certification content | 1. Limited protected time and scheduling conflicts  2. Challenges in selecting and scoping topics  3. Lack of formal outcome data or long-term evaluation |
| [59] Adreak et al. 2021 | Physicians, trainees, and stakeholders | Development project with pilot evaluation (n=51 beta testers) | Design, implementation and evaluation of an online curriculum on women’s cardiovascular health (9 modules, case-based, accredited) | Curriculum developed using evidence and lived experience; beta-testing confirmed usability and relevance; formal evaluation ongoing | Early feedback suggests improved knowledge and engagement; long-term behavioral and clinical outcomes yet to be measured | **Education**  (Multidisciplinary education (university and training)) | 1. Structured 6-step curriculum development process  2. Inclusion of lived experience (patients)  3. Flexible, accredited delivery format (online, asynchronous) | 1. Lack of existing curricula to build on  2. Gaps in physician knowledge and confidence  3. Need for sustained evaluation to assess impact |
| [60] Clever et al. 2020 | Experts from German-speaking universities with implementation experience | 9 expert interviews; qualitative design | Semi-structured interviews on strategies, content, and implementation of sex- and gender-sensitive medicine in curricula | Varied integration models across universities; emphasized importance of faculty involvement, structural anchoring, and longitudinal curriculum integration | Identified success factors and obstacles; provided practice-based insights | **Education**  (Multidisciplinary education (university and training)) | 1. Structural anchoring (e.g. dedicated coordination units and change agent)  2. Combined top-down and bottom-up processes  3. Curricular integration incl. exam relevance | 1. Lack of faculty engagement and expertise  2. Perception as a “women’s issue”  3. Limited resources and sustainability challenges |
| [61] Tannenbaum & Moineau 2016 | None (commentary by institutional leaders) | Not applicable (conceptual commentary) | Discussion of two system-level levers: (1) accreditation standards, (2) student advocacy as drivers for integrating gender medicine in curricula | Identified potential for top-down and bottom-up strategies to create lasting curricular change; highlighted McGill case as example | Provides conceptual and strategic guidance | **Education**  (Multidisciplinary education (university and training)) | 1. Accreditation as enforcement mechanism 2. Mobilization and empowerment of students through bottom-up approach 3. Alignment with broader education reform movements | 1. Gender topics not consistently included in accreditation standards  2. Limited student awareness of influence pathways  3. Fragmented implementation without system-wide coordination |
| [62] Jenkins et al. 2016 | U.S. medical students (MD and DO) | 1,097 participants; national cross-sectional survey | Online survey assessing awareness, attitudes, and curricular exposure to sex- and gender-based medicine (SGBM) | High agreement on the importance of SGBM and its distinction from women’s health; exposure increased with year, but curricular gaps remained | Survey revealed substantial unmet need for structured SGBM education | **Education**  (Multidisciplinary education (university and training)) | 1. Sex and gender curriculum change team  2. Recognized relevance for clinical care  3. Interest in comprehensive integration | 1. Inconsistent curricular inclusion across schools  2. Low self-perceived preparedness for clinical application  3. Disparities in exposure by gender and class year |
| [63] Thande et al. 2019 | U.S. medical school curriculum directors | Survey of 30 U.S. medical schools (cross-sectional study) | Assessment of the extent to which sex and gender are included in medical curricula | Found limited and inconsistent integration of sex/gender topics in curricula | Identified gaps and variation | **Education**  (Multidisciplinary education (university and training)) | 1. Growing awareness of importance of sex/gender in medicine  2. Faculty champions,change agents and curriculum committee  3. Accreditation standards encouraging inclusion and communication | 1. Lack of standardized requirements  2. Insufficient faculty training  3. Low institutional prioritization |
| [64] Böckers et al. 2017 | Medical students | 182 medical students (pilot study) | Implementation of a longitudinal, gender-specific medical curriculum | Identified generally positive attitudes of students towards gender-sensitive teaching; noted sex-specific differences in perception | Showed that female students rated the relevance of gender-sensitive content significantly higher | **Education**  (Multidisciplinary education (university and training)) | 1. Positive attitude among students  2. Integration in existing curriculum  3. Awareness of gender relevance among educators | 1. Limited generalizability due to pilot setting 2. Differences in acceptance between male and female students 3. Initial lack of routine in teaching gender-sensitive content |
| [65] Nachtschatt et al. 2017 | Medical students and educators at Austrian universities. | Survey of medical faculties and students | Assessment of the integration of gender medicine into medical curricula, including curriculum content analysis and surveys to evaluate awareness and implementation. | Gender medicine is only partially integrated into medical education, with significant variability between universities | The integration efforts are still in early stages, with limited impact on medical education practices; there is a need for more structured incorporation. | **Education**  (Medical education and training (university)) | 1. Profit from an exchange of ideas 2. Chair for Gender Medicine  3. Existing guidelines and recommendations promoting gender-sensitive education | 1. Lack of curriculum time and resources  2. Insufficient faculty training on gender medicine, 3. Gender Medicine in medical education varies from region to region |
| [66] Steinböck et al. 2020 | Medical students and lecturers at the Innsbruck and Vienna medical universities. | Comparative analysis of the lecture series, with data collected from 62 lecturers and 11 students | Lecture series on gender medicine as a teaching format, aimed at integrating gender-specific content into medical education. | The study assessed students´ and Teachers´ perspective of the lecture series | The lecture series was generally well-received, leading to increased awareness and knowledge about gender medicine among students. It also enhanced their understanding of gender-specific health issues. | **Education**  (Medical education and training (university)) | 1.Engaging and relevant content tailored to medical education  2.Support from university faculty and administration  3.Interactive teaching methods that encouraged student participation | 1.Limited time within the existing curriculum to incorporate additional content  2.Possible lack of faculty expertise in gender medicine  3.Resistance to curriculum change or integration challenges |
| [67] Hochleitner 2013 | Gender differences in patients with cardiovascular disease | Overview | The article examines gender-specific factors in cardiovascular diseases, including differences in risk factors, clinical presentation, and treatment approaches | Differences in disease presentation, management, and prognosis between genders. | Not applicable | **Education**  (Medical education and training (university)) | 1.Increased awareness of gender differences  2. Gender medicine in guideline of every speciality  3.Tailored treatment approaches for men and women | 1.Lack of gender-specific research data  2.Potential biases in diagnosis and treatment  3.Limited integration of gender considerations into clinical practice |
| [68] Park et al. 2018 | Graduate students enrolled in a course on sex and gender medicine in Korea | 12 students, 10 professors, pre- / post- quantitative study design | Graduate course on sex and gender medicine. | The outcomes involved students' experiences, perceptions, and possibly their learning or attitudes towards sex and gender medicine | Improvement of sex and gender knowledge in medicine (p< 0.001) | **Education**  (Medical education and training (university)) | 1.As part of the fundamental curriculum  2. International networking  3.Development of teaching materials | 1.Lack of prior knowledge or familiarity with the subject  2. Lack of time and recources  3. Lack of evidence-based content and faculty interest |
| [69] Yamazaki et al. 2020 | Faculty members and students involved in medical education. | 43 schools were considered as study targets, of which 33 faculty members belonged to medical education departments and 10 to other departments., survey-based descriptive study with seven-item anonymous questionnaire | The intervention involves the implementation or presence of sexual and gender minority (SGM) education within medical curricula.  Monitoring framework for developing competency-based sexual and gender minority education in Japanese Medical Schools. | The outcomes include the prevalence of SGM education in medical schools and insights into the curriculum content and future directions for such education. | The study suggests that targeted curriculum can improve students' understanding of SGM patients and their healthcare needs. | **Education**  (Medical education and training (university)) | 1. Presence of qualified instructors.  2. Institutional policies supporting SGM education.  3. Integration of SGM topics into existing medical core curriculum | 1.Lack of suitable instructors,  2. Absence of school policy in medical schools,  3. Lack of future plans in teaching SGM |
| [70] Midik et al. 2020 | Medical interns in Turkey | 1739 interns in six medical faculties in four different geographical regions of Turkey, cross-sectional survey | Assessing perceptions or experiences related to gender in medical education | The outcomes relate to interns' perspectives on gender issues in medical education | The students who stated that their gender had not affected their educational lives during clinical training reported that it had adversely impacted their internships (p<0.001) | **Education**  (Medical education and training (university)) | 1.Greater focus on role modeling and purposeful teaching of *gender concepts* from the earliest stages of medical education  2. Involving all hospital personnel in order to prevent gender discrimination  3.Gender awareness must be included in the medical education spiral curriculum | 1.Gender bias is encountered in medical textbooks, medical curricula, and in other education tools  2.Male students are less aware of the concept of gender than female students |
| [71] Ton et al. 2016 | Faculty members and educators | The study utilized a daylong retreat to design a 4-year sexual orientation and gender identity (SOGI) curriculum. Participants completed pre- and post-surveys to assess their perspectives and knowledge. | The retreat focused on developing a structured SOGI curriculum for medical students, incorporating expert discussions and curriculum database reviews. | Participants showed significant improvements in their confidence in creating an SOGI curriculum, knowledge of teaching resources, and understanding of where SOGI competencies should be integrated. | A 6-month follow-up survey indicated strong ongoing support for the new curriculum, though challenges in implementation remained. | **Education**  (Medical education and training (university)) | 1. Expert involvement in curriculum development.  2. Institutional support for integrating SOGI topics.  3. Structured approach using a retreat model to design the curriculum. | 1. Limited faculty expertise in SOGI education.  2. Challenges in curriculum implementation despite initial enthusiasm.  3. Variability in institutional commitment to sustaining the curriculum. |
| [72] Eisenberg et al. 2013 | Final-year medical students undertaking a Women's Health rotation | The study surveyed students (n= 30) to assess their gender awareness in medical education. | The research examined how gender awareness is integrated into the curriculum and how students perceive its importance. | The study highlights the level of gender awareness among medical students and the impact of the Women's Health rotation on their perspectives. | There was a significant difference in gender sensitivity between the students who received one week of WH teaching when compared to those who did not (*p* < 0.05). | **Education**  (Medical education and training (university)) | 1. Improving gender awareness | Not applicable |
| [73] Dijkstra et al. 2008 | Medical textbooks used in Dutch medical schools. | 11 textbooks were screened, covering internal medicine/cardiology, psychiatry, and pharmacology | The study examined the availability and accessibility of gender-specific knowledge in medical textbooks | Gender-specific information was scarce or absent, with most references limited to epidemiological data and reproductive health | The study concluded that gender bias in textbooks may lead to future doctors being unaware of relevant gender differences in diagnosis, treatment, and disease presentation | **Education**  (Medical education and training (university)) | 1. Encourage medical students to be critical with literature  2. Inclusion of gender-sensitive content in textbooks  3. Awareness among educators about gender bias in medical literature | 1. Current medical textbooks are still gender-biased  2. Future doctors will be unaware  3. Traditional medical assumptions treating male and female bodies as generally the same |
| [74] Henrich et al. 2008 | The participants were third and fourth-year medical students at U.S. allopathic medical schools. | The study included 1,267 students from 101 of the 125 U.S. medical schools; survey-based study with 101 items | The intervention involved an online survey conducted by the American Medical Women’s Association (AMWA) to assess students' perceptions of the adequacy of women’s health and sex/gender-specific teaching and their preparedness to care for female patients. | The study found that medical students reported moderate preparedness to care for women but rated the curriculum coverage of women’s health and sex/gender-specific topics as brief to moderate. | The intervention effect showed that students felt moderately prepared to perform clinical skills related to women’s health, but there were significant gaps in the curriculum coverage of women’s health and sex/gender-specific topics. | **Education**  (Medical education and training (university)) | 1. Patient Education: Providing education about women’s health and sex/gender-specific topics.  2. Regular Monitoring: Continuous assessment and feedback on students' preparedness and curriculum coverage.  3. Multidisciplinary Approach: Involvement of various departments and disciplines in teaching women’s health. | 1. Curriculum Gaps: Inadequate coverage of women’s health and sex/gender-specific topics in the curriculum.  2. Resource Availability: Limited resources and faculty knowledgeable about women’s health.  3. Institutional Resistance: Traditional views and resistance to integrating controversial topics into the curriculum. |
| [75] Seeland et al. 2016 | Medical students and health professionals from Europe | More than 90 users registered for the eGender Medicine learning modules; web-based interactive knowledge-sharing platform study. | The intervention involved the development and implementation of the eGender platform. The platform included e-learning modules focusing on basic knowledge and specific medical disciplines, with tools for online communication and collaborative work. | The eGender platform was flexible and user-friendly, providing evidence-based high-quality learning material. | The eGender platform successfully provided comprehensive and high-quality educational material on sex- and gender-specific medical education, which was well-received by the users. | **Education**  (Medical education and training (university)) | 1. Patient Education: Comprehensive educational material  2. Regular Monitoring: Continuous assessment and feedback on the platform's effectiveness and user engagement.  3. Multidisciplinary Approach: Involvement of experts from various universities and disciplines in developing the learning materials. | 1. Curriculum Gaps: Initial lack of comprehensive sex- and gender-specific content in medical curricula.  2. Limited resources and faculty knowledge about sex- and gender-specific medicine.  3. Institutional Resistance: Challenges in integrating the platform into existing medical education systems. |
| [76] Dielissen et al. 2012 | Experts in the field of gender medicine education and doctor–patient communication. | 59 participants (28 gender medicine education experts and 31 doctor–patient communication experts); three-round Delphi study with questionnaires to define and validate gender criteria | Development of gender criteria for inclusion in communication skills assessment | Four gender criteria achieved consensus after the third round. These criteria were considered important for inclusion in communication skills assessment. | The inclusion of gender criteria in communication skills assessment was rated as important and feasible, promoting better communication between doctors and patients. | **Education**  (Medical education and training (university)) | 1.The enhanced gender criteria help-teaching faculty relate more easily to gender-sensitive communication skills feedback and assessment  2.Can help to identify curricular deficiencies in teaching communication skills | 1. Implementation challenges  2. Gender stereotyping |
| [77] Rojek & Jenkins 2016 | Medical students and faculty members involved in medical education. | Review and discussion of the implementation of sex- and gender-based medicine (SGBM) in medical education. | The intervention involved integrating a sex- and gender-based medicine lens into medical education curricula and evaluated students´ knowledge and competencies | Improved students' understanding of sex and gender differences in health and disease. | The intervention effect was positive, leading to enhanced awareness and knowledge among medical students regarding sex and gender differences in medical practice. | **Education**  (Medical education and training (university)) | 1. Leaders should be involved in curricula change process  2. Availability of educational resources and materials.  3. Inclusion of SGBM topics in the curriculum. | 1. Resistance to curriculum changes.  2. Limited faculty expertise in SGBM.  3. Insufficient time allocated for SGBM topics in the curriculum. |
| [78] Verdonk et al. 2009 | Medical educators and change agents involved in medical education. | Qualitative study involving semi-structured interviews with 18 participants | Integration of gender mainstreaming into medical education curricula. | The study found that gender mainstreaming in medical education led to increased awareness and understanding of gender issues among medical educators and students. | Enhanced awareness and knowledge regarding gender issues in medical practice. | **Education**  (Medical education and training (university)) | 1. Change agents  2. Train-the-trainers course  3. Inclusion of gender topics in the curriculum (gender-specific education materials) | 1. White male curriculum developers lack the view  2. Limited faculty expertise and knowledge in gender issues.  3. Insufficient time allocated for gender topics in the curriculum. |
| [79] van der Meulen et al. 2017 | Medical educators and coordinators involved in the Nijmegen medical curriculum. | 8 course coordinators;  Study Design: Longitudinal evaluation and qualitative study involving interviews. | Screening coursebooks for gender perspective | Sustained implementation of gender health issues in the curriculum over the past decade, with additional changes made. | Positive effect, leading to an ongoing gender perspective in the medical curriculum. | **Education**  (Medical education and training (university)) | 1. Gender-sensitive attitude of coordinators and teachers.  2. Presence of sex and gender in learning objectives and evaluations.  3. Organizational support and curriculum revisions. | 1. Other priorities in the curriculum  2. Limited time allocated for sex and gender topics.  3. Resistance to curriculum changes. |
| [80] Weyers et al.2017 | Medical students and teachers. | The study involved 5 teachers and 247 fourth-semester medical students; qualitative methods in a student focus group and quantitative methods in a seminar. | The intervention involved the adaptation and implementation of the German "Gender Lens" tool to introduce gender medicine to medical students. | The study found that the Gender Lens tool provided a useful framework for analyzing sex and gender differences in medical education. | Positive effect for introducing gender medicine | **Education**  (Medical education and training (university)) | 1. Support from faculty  2. Comprehensive educational material | 1.Initial resistance to new teaching methods.  2.Limited time for implementation  3.Variability in teacher engagement. |
| [81] Cheng & Yang 2015 | Medical students at a Taiwanese medical school | 30 medical students; Qualitative study using focus groups and interviews | Examination of the hidden curriculum related to gender issues in medical education. | The study found that the hidden curriculum perpetuated gender stereotypes and biases, affecting students' perceptions and interactions. | The intervention highlighted the need for addressing gender biases and stereotypes in medical education to promote a more inclusive environment. | **Education**  (Medical education and training (university)) | 1.Awareness of gender issues among students.  2.Promote gender sensitivity in the use of language in class by teachers and in extracurricular activities by students  3.Inclusion of gender topics and LGBT in formal curriculum. | 1.Perpetuation of gender stereotypes.  2.Resistance to change in institutional culture.  3.Unequal gender ratio and the perceived dominance of masculine culture in the medical profession |
| [82] Safdar et al. 2019 | Health professionals and educators from several disciplines. | Workshop proceedings and qualitative analysis. | Integration of sex and gender concepts into an interprofessional curriculum. | The workshops led to the development of strategies and recommendations for integrating sex and gender health education into interprofessional curricula. | The intervention was effective in raising awareness and providing actionable strategies for incorporating sex and gender concepts into health education. | **Education**  (Medical education and training (university)) | 1.Collaborative environment.  2.Support from institutional leadership.  3.Availability of educational resources. | 1.Resistance to change.  2.Limited faculty expertise.  3.Time constraints in curricula. |
| [83] Tollemache et al. 2021 | Course leads at UK medical schools | Responses were received from 19 out of 37 institutions (response rate: 51%); Cross-sectional online survey with 30 questions | The survey aimed to review the content of LGBT teaching within the curricula of UK medical schools. | The study found significant variation in the amount and breadth of LGBT content within the undergraduate curricula of UK medical schools. | The intervention highlighted the need for increasing the quantity and quality of LGBT content in medical education. | **Education**  (Medical education and training (university)) | 1.Institutional support for LGBT teaching (lead role for LGBT)  2.Positive attitudes towards LGBT inclusion  3.Internal and external collaborations  3.Innovative content delivery | 1.Lack of space and time within the curriculum.  2.Limited faculty expertise in LGBT health (no specific responsibility)  3.Resistance to curriculum changes. |
| [84] Ludwig et al. 2015 | Medical educators and students at Charité – Universitätsmedizin Berlin. | Case study of curriculum development and implementation. | Development of a 10-step approach to incorporate sex and gender medicine into the curriculum, including lectures, seminars, and practical courses. | The integration led to the inclusion of sex and gender perspectives in 5% of the learning objectives and assessments. | The intervention was successful in systematically integrating sex and gender medicine into the curriculum, enhancing students' knowledge and skills. | **Education**  (Medical education and training (university)) | 1.Support from faculty and administration.  2.Presence of a change agent.  3.Systematic approach to curriculum development. | 1.Resistance to curriculum changes.  2.Limited time for implementation.  3.Variability in faculty engagement. |
| [85] van Leerdam et al. 2014 | Medical students from Radboud University, Nijmegen, The Netherlands. | 29 medical students; Qualitative study using focus groups | Examination of gender-based education during clerkships. | The study found that clinical teachers rarely discussed gender differences during clerkships, leading to students feeling insufficiently prepared to become gender-sensitive doctors. | The intervention highlighted the need for improved gender-based education during clerkships to enhance students' competencies in gender-sensitive medical practice. | **Education**  (Medical education and training (university)) | 1.Awareness of gender issues among students.  2.Female physicians were more likely to take gender differences in account  3.Inclusion of gender topics in formal curriculum. | 1.Insufficient knowledge among clinical teachers.  2.Unawareness of gender differences (stereotyping).  3.Teachers´ weak interest |
| [86] Risberg et al. 2009 | Theoretical model analysis | Theoretical model development and analysis | Development of a three-step theoretical model to understand and analyze gender bias in medicine. | The model illustrates that gender bias can arise from assuming sameness and/or equity between women and men when there are genuine differences | The model provides a conceptual framework for discussing and avoiding gender bias in clinical work, medical education, career opportunities, and healthcare policies. | **Education**  (Medical education and training (university)) | 1.Awareness of gender issues.  2.Continuous reflection on gender attitudes.  3.Consciousness-raising activities among students, teachers, researchers, and decision-makers. | 1.Unawareness of gendered attitudes.  2.Gendered stereotypes.  3.Resistance to change in institutional culture. |
| [87] Seeland et al. 2019 | Medical educators and students involved in gastroenterology and visceral surgery courses. | Evaluation of primary and secondary literature on the integration of gender-sensitive teaching content. | Integration of gender-sensitive teaching content into medical curricula. | Increased visibility and implementation of gender-sensitive teaching content in medical curricula, particularly in gastroenterology and visceral surgery. | Positive effect, leading to enhanced awareness and knowledge of gender differences in medical practice among students and educators. | **Education**  (Medical education and training (university)) | 1.Increased visibility of gender medicine at conferences and in publications.  2. Support from institutional leadership.  3.Gender content in curricula | 1.Lack of a nationwide concept for integrating gender-sensitive content.  2.Variable knowledge levels among students depending on the university. |
| [88] Encandela et al. 2019 | Medical educators and students involved in the development and implementation of the curriculum. | Case study and qualitative analysis. | Development and implementation of an integrated curricular sequence (workshops, lectures, and interactive sessions) focusing on sexual and gender minority (SGM) health. | The curriculum sequence led to increased awareness and understanding of SGM health issues among medical students. | Positive effect, with students demonstrating improved knowledge and competencies in SGM health. | **Education**  (Medical education and training (university)) | 1.Curriculum development  2.Collaboration among faculty members  3.Availability of educational resources and materials | 1.Resistance to curriculum changes  2.Limited faculty expertise in SGM health  3.Time constraints within the existing curriculum |
| [89] Gaida et al. 2020 | Medical students (mentees) and faculty members at Leipzig University. | 108 medical students participated in the elective; Project report | Implementation of an elective course on career management for medical students, focusing on gender sensitivity. | The elective course successfully promoted gender-sensitive teaching and contributed to the development of gender sensitivity at Leipzig University. | Positive effect, leading to increased awareness and understanding of gender issues among medical students and faculty members. | **Education**  (Medical education and training (university)) | 1.Female students and professors promote gender equality  2.Support from institutional leadership.  3.Availability of educational resources and materials. | 1.Resistance to curriculum changes.  2.Limited faculty expertise in gender sensitivity.  3.Time constraints within the existing curriculum. |
| [90] Pai et al. 2021 | Nursing students | 58 nursing students were included in pre- and post-observation (cohort study) | A situation model nursing education action program aimed at increasing gender-bias awareness and reducing gender-friendliness barriers. | Levels of gender-bias awareness and the identification of gender-friendliness barriers among the participants. | Mean student scores showed that gender-bias awareness was significantly decreased at Week 2 (p < .001) and Week 4 (p < .001) and that mean gender-friendliness barrier scores significantly declined at Week 4 (p < .001). | **Education**  (Nursing education) | 1.Faculty professional learning (community workshop)  2. Promote gender awareness and friendliness among nurses | Not applicable |
| [91] Noonan et al. 2021 | Transgender and genderqueer standardized patients | The study involved 10 participants and utilized semi-structured focus groups | The intervention involved the use of standardized patient simulation to teach gender-affirming clinical skills | Participants' experiences and perceptions of simulation and gender-affirming care | Participants felt empowered and hopeful about the impact of their participation on future medical care for gender minorities. | **Education**  (Nursing education / LGBTQ+ education and care) | 1. Training promotes GSC+ care  2.Enhanced intereation with gender minorities | 1.Communication gaps  2.Minorities are underrepresented in medical education  3. Students lack foundational SGM knowledge |
| [92] Lindsay & Kolne 2020 | Pediatric rehabilitation healthcare providers | The study involved 23 participants (19 women, 3 men, 1 transgender man) and used an interpretive descriptive qualitative design | The intervention focused on identifying and describing the training needs for gender-sensitive care among pediatric rehabilitation healthcare providers | Participants' knowledge about gender-sensitive care, the content of the desired training, and the preferred delivery methods for the training | Lack of knowledge about gender-sensitive care and the need for more training. It also identified the specific content and delivery methods desired by healthcare providers | **Education**  (Nursing education / Institutions of care (medical) patient record) | 1.Suportive organizational culture  2.Interdisciplinary collaboration  3.Ongoing education on equity, diversity and inclusion | 1.GSC+ is not a priority  2. Binary documentation of patients  3.Resistance to change and gender-based discrimination |
| [93] Klotzbaugh et al. 2020 | Advanced practice nursing students | The study involved 27 participants and utilized a pre- and post-test design | The intervention was a gender minority health education module | Knowledge of medical guidelines, disparities, policies, and attitudes specific to gender minorities | The study demonstrated statistically significant improvement in knowledge and attitudes towards gender minority health care | **Education**  (Nursing education) | 1.Readiness and willingness of nursing faculty to invest the time to integrate concepts of gender minority health into their coursework  2.Cultural competency module | Not applicable |
| [94] Hsieh et al. 2013 | Internal medicine residents | The study involved 100 residents (64% response rate from 156 residents) and used a 67-item questionnaire | Assessing residents' comfort levels, perceived adequacy of training, and frequency of managing 13 core women's health topics | Comfort levels in managing women's health topics, perceived adequacy of training, and frequency of managing these topics | The study found that the majority of residents reported low comfort levels. Linear relationship between low comfort levels and limited training opportunities, and low comfort levels and low frequency of managing women's health topics | **Education**  (Medical education for physicians (postgraduate)) | 1.Women´s health curriculum  2.preexisting infrastructure (such as  preventive health screening protocols) can help generate important and sustainable training opportunities  3.Investing in faculty development | Not applicable |
| [95] Farkas et al. 2018 | Women's health track (WHT) graduates from three different residency program | The study involved 29 WHT graduates and used a cross-sectional survey design | The intervention was the women's health residency track, which aimed to provide specialized training in women's health | The outcomes measured included career involvement in women's health, leadership roles, clinical practice, scholarship, and teaching | Graduates of the women's health residency tracks were more likely to assume women's health leadership roles (34.5% vs. 0.0%, p-value 0.018) and remain involved in women's health in various capacities | **Education**  (Medical education for physicians (postgraduate)) | 1.Women´s health track enhance residents´ awareness  2.Strong female mentorship  3.Balancing career in academic medicine and family life | Not applicable |
| [96] Kling et al. 2016 | Post-graduate medical residents | The study involved 271 residents and used a cross-sectional survey design | Assess current knowledge of sex- and gender-based medicine and identify barriers and preferred teaching methods for addressing sex and gender issues in health and disease | Residents' knowledge of sex- and gender-based medicine, their comfort in managing sex- and gender-related health issues, and their perceptions of the adequacy of their training | The study found significant knowledge gaps in sex- and gender-based medicine among residents. Many trainees did not understand the potential impact of sex and gender on clinical practice and believed it did not pertain to their specialty | **Education**  (Medical education for physicians (postgraduate)) | 1.Supportive organizational culture  2.Faculty development  3.Possibility to integrate into existing curricula | 1. Lack of awareness  2. Limited time  3.Perceived irrelevance and resistance of change |
| [97] Dielissen et al. 2014 | General practitioner (GP) trainees in the Netherlands. | 207 GP trainees. Prospective cohort study with questionnaire survey | Comparison of two gender medicine teaching methods: a modular approach and a mainstream approach. | Significant difference in change in gender knowledge scores between the modular cohort compared with the mainstream and control cohorts. | The modular teaching method was not found to be more favorable than the mainstream method for teaching gender medicine in GP training. | **Education**  (Medical education for physicians (postgraduate)) | 1.Structured teaching methods.  2.High response rates.  3.Inclusion of gender awareness and knowledge assessments. | 1.Female GP trainees demonstrated higher gender awareness than male GP trainees.  2.Variability in gender awareness among trainees. |
| [98] Dielissen et al. 2009 | General practitioner (GP) trainees in the Netherlands. | Pre-test/post-test study with a non-randomized, voluntary-group of nine third-year residents and nine GP-trainers and control group with 19 third-year residents and 19 GP-trainers | Two supplementary tutorials of 3 hours each, over a 6-month period to teach residents gender awareness. | No significant effect could be demonstrated on the intervention group’s stereotypes towards patients (mean score increased from 2.33 to 2.35; P = 0.72) or towards doctors (mean score increased from 2.52 to 2.55; P = 0.82). | Participants’ knowledge of gender-specific medicine increased | **Education**  (Medical education for physicians (postgraduate)) | Not applicable | Not applicable |
| [99] Schreitmüller et al. 2018 | Medical students, lecturers, physicians, and the public | 30 participants in focus groups and 149 students in a quantitative survey using an online questionnaire | Development and implementation of the "GenderMed-Wiki" online platform to provide knowledge on sex and gender aspects in medicine | The platform was found to be suitable for providing knowledge on sex and gender aspects in medicine, with areas identified for further optimization. | Positive effect, leading to increased awareness and understanding of sex and gender aspects in medicine among users. | **Education**  (Medical education for physicians (postgraduate)) | 1.Topic in exams  2.Change agent  3.Collaboration among various universities and institutions. | 1.Usability challenges. |
| [100] Bönte et al. 2008 | Primary care physicians from the United States, the United Kingdom, and Germany. | 384 physicians (128 from each country) in a factorial experiment using videotaped patient consultations. | Professional actors played the role of patients with symptoms of coronary heart disease (CHD). Several versions of the same script were taped, featuring patient-actors of different gender, age, race, and socioeconomic status. Physicians were asked how they would diagnose and treat the patient after viewing the video. | The study investigated whether and to what extent doctors' diagnostic and therapeutic decisions in CHD were influenced by patient gender. | Gender influenced diagnostic strategies more than treatment decisions, with variations across countries. | **Education**  (Medical education for physicians (postgraduate)) | Not applicable | 1.Variability in physicians' interpretation of symptoms based on patient gender  2.Differences in healthcare systems and practices across the three countries  3.Female patients receive less questioning and were less likely to receive accurate diagnosis |
| [101] Celik et al. 2008 | Healthcare professionals from mental health, hospital, and nursing home settings. | Nine in-depth, semi-structured interviews and three focus groups composed 8-12 healthcare professionals. | Investigated the extent of diversity in healthcare practices and explored barriers and opportunities for implementation. | Diversity is recognized as important but often reduced to one dimension (sex), with a neutral, disease-oriented approach remaining dominant. | Identified barriers and opportunities for implementing diversity in healthcare practices. | **Education**  (Medical education for physicians (postgraduate)) | 1.Supportive political climate  2.Emerging sense of urgency to address diversity.  3.Development of good practice | 1.Lack of awareness and knowledge about diversity.  2.Poor information and communication.  3.Organizational constraints. |
| [102] Celik et al. 2009 | General Practitioners (GPs) | Nine semi-structured interviews conducted among nine pairs of GPs | Investigated the facilitators and barriers to maintaining gender sensitivity in family practice. | Identified factors influencing the extent to which GPs maintain a gender-sensitive approach in their practice | Highlighted the importance of gender awareness, repetition, reminders, motivation triggers, and professional guidelines in facilitating gender sensitivity | **Education**  (Medical education for physicians (postgraduate)) | 1.Gender awareness.  2.Professional guidelines.  3.Motivation triggers. | 1.Lack of skills and routines.  2.Scepticism by GPs  3.Heavy workload. |
| [103] Walter 2021 | Medical students and emergency medicine (EM) residents | 12 trainees and 11 medical students, pre- and post-course surveys were obtained. Surveys were designed to assess awareness, knowledge, and attitudes regarding Sex- and Gender-based Medicine (SGM) | Introduction of a unique elective course on sex and gender medicine in emergency medicine | To close the knowledge and curricular gap regarding sex and gender differences in emergency medicine | Enhanced understanding and awareness of sex and gender differences in disease presentation, treatment, and outcomes among participants | **Education**  (Medical education for physicians (postgraduate)) | 1.Different learning modules as journal clubs, formal didactic sessions, flipped classroom video and readin assignments or case scenarios enhance physicians´ EM competency on SGM | Not applicable |
| [104] Ashurst et al. 2014 | Emergency medicine residents | The study design involved reviewing existing educational models and proposing guidelines for incorporating gender-specific education | Implementation of gender-specific education in emergency medicine training programs through various educational strategies such as curriculum development, grand rounds, simulation, bedside teaching, and journal clubs | The goal was to improve knowledge and attitudes towards gender differences in emergency medicine, ultimately enhancing patient care | The proposed guidelines aimed to systematically incorporate sex and gender health concepts into medical education, postgraduate training, and continuing medical education (CME) | **Education**  (Medical education for physicians (postgraduate)) | 1.Engagement of educators, administrators, and professional societies.  2.Equal numbers of cases should be mals and femal.  3.Interprofessional and interdisciplinary collaborations. | 1.Lack of qualified educators in sex and gender medicine.  2.Absence of sufficient educational resources (qualified educators) and standardized curricula.  3.Challenges in updating medical textbooks and integrating new knowledge into clinical rotations. |
| [105] Willging et al. 2019 | Transgender and gender non-conforming (TGGNC) patients, physicians, nurses, and non-clinical staff | 31 semi-structured interviews with 11 TGGNC patients, 6 physicians, 7 nurses, and 7 non-clinical staff | Examined experiences and service delivery in Emergency Departments (EDs) for TGGNC patients | Identified structural issues impacting the health and wellbeing of TGGNC patients and service delivery practices in EDs | Highlighted the need for training ED personnel and modifying practice settings to ensure appropriate services for TGGNC patients | **Education**  (Medical education for physicians (postgraduate)) | 1.Respect and humanity in patient interactions.  2.Training and preparation of providers and staff.  3.Supportive communication | 1.Lack of resources for structural prescriptions.  2.Spatial considerations in EDs.  3.Insufficient understanding of gender identity and sex at birth. |
| [106] Greenberg et al. 2023 | Women seeking personalized preventive health recommendations | HeaRT was used 13,749 times by 12,547 users between May 2018 and July 2021. The study involved analyzing usage patterns and engagement through web-tool data | Development and implementation of the HeaRT web tool to provide personalized preventive health recommendations for women | Improved engagement in preventive health behaviors among women, with 68.6% of users accessing results and approximately 15% printing or emailing the recommendations | Enhanced awareness and adherence to personalized preventive health recommendations among users, with significant engagement in categories like nutrition, risk factors, and physical activity | **Education**  (Patient-empoewerment) | 1.Promote health literacy  2.Tailored content based on user profiles  3.Integration of evidence-based guidelines and health literacy principles | 1.Lack of socio-economic data collection to fully describe the sample population.  2.Limited tracking of user behavior changes post-usage.  3.Focus on women only, requiring adaptation for male users. |
| [107] Sherman et al. 2021 | Pre-licensure nursing students | The study involved a self-administered online survey with pre- and post-intervention assessments. The exact sample size was not specified in the search results | Integration of an evidence-based curriculum specific to transgender and gender diverse (TGD) health into five nursing pre-licensure courses | Assessed the preliminary efficacy and feasibility of the curriculum in improving TGD-related health knowledge and attitudes among nursing students | Improved students' gender sensitivity, self-reported skills in providing care for TGD people, and knowledge of additional TGD-specific resources | **Education**  (LGBTQ+ education and care) | 1.Engagement and support from educators and administrators.  2.Positive student feedback and increased demand for more TGD content | 1.Limited time allocated to TGD-related content in the curriculum.  2.Providers feelings of uncertainty and even apathy toward the unique needs of TGD people  3.Further research is needed to elucidate  the best educational practices |
| [108] Hana et al. 2021 | Medical students and educators | Overview and recommendations for integrating transgender health into medical curricula. | Integration of transgender health education into medical school curricula, including cultural humility and anti-oppression training, involvement of transgender and gender-diverse community members, and practice-focused training | Improved understanding and awareness of transgender health issues among medical students and educators. | Enhanced ability of future healthcare providers to address the specific health needs of transgender and gender-diverse individuals | **Education**  (LGBTQ+ education and care) | 1.Cultural humility and anti-oppression training.  2.Involvement of transgender and gender-diverse community members.  3.Integration of transgender health into existing curricula. | 1.Lack of qualified educators in transgender health.  2.Insufficient educational resources and standardized curricula.  3.Minimal or no inclusion of transgender health in undergraduate medicine |
| [109] Arthur et al. 2021 | Medical students | Cross-sectional 28-item survey with 252 analyzed responses from 776 eligible participants | Survey exploring views on the undergraduate medical curriculum, awareness of LGBT health issues, attitudes towards LGBT patients, and confidence in providing care | Positive attitudes towards LGBT patients, but variable awareness and confidence. Confidence in discussing sexual orientation increased with the year of study, but not for gender identity | Highlighted the need for increased LGBT-specific training in medical curricula to improve doctor-patient interactions and healthcare quality | **Education**  (LGBTQ+ education and care) | 1.Positive attitudes towards LGBT patients.  2.Desire for more LGBT-specific training and topics in curriculum  3.Knowledge and awareness of LGBT specific health issues | 1.Lack of specific training on LGBT health needs  2.Variable confidence in discussing gender identity.  3.Existing curricular structures not adequately addressing LGBT health issues |
| [110] Persson Tholin & Broström 2018 | Self-identified transgender or gender-diverse individuals | Semi-structured interviews with 12 participants | Explored experiences of accessing non-transition-related health care in Sweden | Participants experienced difficulties in having their gender identity acknowledged by healthcare staff. Many healthcare professionals lacked knowledge about transgender people, leading to participants taking responsibility for ensuring adequate care | Negative experiences or fear of them led some participants to delay seeking health care or to withhold their transgender identity during consultations | **Education**  (LGBTQ+ education and care) | 1.Use right names and pronouns for transgender  2. Training and preparation of providers and staff  3.Implementation of transgender needs in nursing and medical school curricula | 1.Lack of resources for structural prescriptions.  2.Spatial considerations in healthcare settings.  3.Insufficient understanding of gender identity |
| [111] Kelley et al. 2008 | Second-year medical students, University of California, San Francisco | Cross-sectional survey with a 140 students, pre- and post-intervention questionnaires (16 statements) to assess knowledge, attitudes, and beliefs | Three-part intervention composed of a syllabus, a 1-hr patient  panel, and a 1-hr small-group session focusing on case studies | Indicating increased knowledge about LGBT health issues, willingness to treat patients with gender identity issues, and awareness of the clinical relevance of sexual identity and practices. | Significant short-term improvements in students' knowledge and beliefs about LGBT health | **Education**  (LGBTQ+ education and care) | 1.Supportive faculty and administration.  2.Evidence-based curriculum development.  3.Positive student feedback and engagement. | 1.Limited time within the curriculum to cover LGBT health comprehensively.  2.Initial lack of knowledge and sensitivity among students.  3.Resistance to change in established educational practices. |
| [112] Ufomata et al. 2018 | Internal medicine residents and faculty preceptors. | 153 residents and 35 faculty preceptors were eligible to participate. Surveys were conducted pre- and post-intervention. | A four-module, case-based, interactive curriculum on LGBT primary care, covering topics such as understanding LGBT issues, cultural competencies, health promotion, disease prevention, mental health, violence, and reproductive health | Improved knowledge and confidence in providing primary care to LGBT patients among residents | Significant improvements in knowledge (from 42% to 66% correct answers) and confidence in various aspects of LGBT primary care | **Education**  (LGBTQ+ education and care) | 1.Engagement of general medicine faculty.  2.Use of case-based, interactive modules.  3.High baseline perceived importance of LGBT topics | 1.Lack of qualified educators in LGBT health.  2.Limited time within the curriculum teaching.  3.Challenges in integrating new content into established curricula |
| [113] Müller 2013 | Academic staff at the University of Cape Town's Faculty of Health Sciences | 127 academics responded to the survey, with 93 completing the questionnaire. The study involved a curriculum mapping exercise through an online survey | The survey aimed to determine the extent of LGBT health-related content, pedagogical methodology, and assessment in the medical curriculum | The study found that LGBT health-related content was largely discretionary, unsystematic, and not incorporated into the overarching curriculum structure. Key LGBT health topics such as safer sex, mental health, substance abuse, and adolescent health were not adequately addressed | Highlighted the need for coordinated initiatives to integrate LGBT health-related content into all health sciences curricula | **Education**  (LGBTQ+ education and care) | 1.Recognition of the need for LGBT-specific health care in recent South African policies.  2.Support for coordinated initiatives to integrate LGBT health content.  3.Emphasis on developing professional attitudes and behavior concerning care for LGBT patients. | 1.Lack of systematic incorporation of LGBT health content into the curriculum.  2.No opportunity for students to challenge their own attitudes towards LGBT patients.  3.Key LGBT health topics not being addressed in the curriculum. |
| [114] Smith et al. 2021 | Social work and nursing students | 58 social work and nursing students participated in a mixed-methods study with pre- and post-surveys | An interdisciplinary curriculum developed by social work and nursing faculty, including lectures, film clips, discussion points, and question-and-answer exercises | The intervention aimed to improve cultural awareness and knowledge of LGBT older adults' health and quality of life | Statistically significant improvements in students' attitudes and knowledge about LGBT older adults' health. The mean correct response rate for factual questions increased from 57.0% pre-intervention to 83.1% post-intervention | **Education**  (LGBTQ+ education and care) | 1.Interdisciplinary collaboration between social work and nursing faculty.  2.Engagement with LGBT older community members in curriculum development  3.Empowered clients to speak without fear of discrimination | 1.Initial lack of knowledge and sensitivity among students.  2.Limited time within the curriculum to teach LGBT health comprehensively.  3.Challenges in integrating new content into established curricula. |
| [115] MacKinnon et al. 2020 | Clinicians, clinician-educators, transgender patients, and clinical care administrators | 22 participants, institutional ethnography study | The study examined how standardized assessment protocols in gender-affirming medicine serve as a form of curriculum and how these protocols influence practice, learning, and teaching | The study identified that practicing gender-affirming medicine is strictly dictated by standardized assessment protocols, which serve as a form of curriculum. | Using assessment protocols enhances teaching and learning | **Education**  (LGBTQ+ education and care) | 1.Engagement of clinicians and clinician-educators  2.Practicing of gender-affirming medicine is strictly dictated by standardized assessment protocols | 1.Limited flexibility in adapting protocols to individual patient needs.  2.Challenges in balancing protocol adherence with advocacy for equitable care.  3.(Trans) people’s access to gender-affirming medicine is limited |
| [116] Rider et al. 2019 | Nurses and physicians who work with adolescents | Semi-structured interviews with 14 participants (nurses and physicians) | The study examined healthcare providers' experiences and attitudes about working with transgender and gender-diverse (TGD) youth to identify specific training needs | Identified multiple opportunities to improve provider education and care experiences of TGD youth | Highlighted the need for specific training to help providers manage discomfort with gender-related topics and develop their knowledge and skills for discussing gender issues | **Education**  (LGBTQ+ education and care) | 1.Talking with patients about gender  2.Availability of resources and training materials.  3.Positive attitudes towards learning about TGD health. | 1.Discomfort with gender-related topics.  2.Lack of training and resources.  3.Institutional and systemic barriers. |
| [117] Scholte et al. 2020 | Successful applicants for medical school | Successful applicants (n= 50); Explorative thematic document analysis of educational assignments | Participants watched a gender-sensitive video resembling a future practical experience (a consultation with a patient) and then formulated their own study plan based on the video | Medical students believed in better care for all patients and thought doctors should obtain gender competences during their medical training | Students preferred to start with acquiring basic biomedical knowledge about differences between men and women and continue their training by developing gender-sensitive communication skills in (simulated) practical settings. | **Education**  (LGBTQ+ education and care) | 1.Teachers as important role models  2. Gender-sensitive video supports students´ learning | 1.Different interpretations of gender-sensitive information by students.  2.Generalization of potential differences to all men and all women  3.Professionals tend to be sceptical about the importance of  gender in health care |
| [118] Snelgrove et al. 2012 | Physicians from Ontario, Canada | 13 physician participants; Qualitative study using semi-structured interviews and analyzed using an emergent grounded theory approach. | Interviews were conducted to capture physicians' perceptions of barriers to providing healthcare for transgender patients. | Physicians identified multiple barriers to providing healthcare for transgender patients, including accessing resources, medical knowledge deficits, ethical considerations regarding transition-related medical care, diagnosing versus pathologizing trans patients, and health system determinants. | Barriers perceived by physicians, emphasizing the need for increased awareness of clinical guidelines and inclusion of transgender health issues in medical education. | **Education**  (LGBTQ+ education and care) | 1.Increased awareness of clinical guidelines for transgender healthcare  2.Support for both trans-focused and trans-friendly primary care models  3.Network may reduce barriers | 1.Lack of medical knowledge regarding transgender healthcare and curriculum  2.Ethical considerations and unfamiliarity with transition-related medical care |
| [119] Fallin-Bennett 2015 | Medical professionals and educators | Commentary / Overview | Overview of disparities in LGBT health and discusses the implications of implicit and explicit biases among medical professionals. It emphasizes the role of the hidden curriculum in perpetuating these biases | The commentary highlights that implicit biases against LGBT individuals among medical professionals can create a cycle that reinforces a professional climate of bias. It underscores the need for awareness and education to address these biases | The overview suggests that raising awareness of LGBT discrimination, increasing exposure to LGBT individuals as colleagues and role models, and modifying medical education curricula can help break the cycle of implicit bias in medicine | **Education**  (LGBTQ+ education and care) | 1.Raising awareness of LGBT discrimination.  2.Increasing exposure to LGBT individuals as colleagues and role models.  3.Modifying medical education curricula to include LGBT health issues. | 1.The hidden curriculum in academic health centers perpetuates bias.  2.Lack of awareness and education about LGBT health issues.  3.Providers feel unprepared and may express stigma or discriminate LGBTQ+ people |
| [120] Yang 2019 | Medical students and teachers | 19 medical students and 2 teachers; Qualitative case-study methodology using in-depth interviews and thematic analysis. | The study designed an LGBT+ Health and Medical Care course in a medical school, incorporating game-based teaching activities to promote gender competency. Feedback was collected through in-depth interviews. | The findings indicated that game-based teaching encouraged student participation and benefited gender knowledge transmission and transformation through competency learning. | Achieving the learning goals of integrating knowledge, attitudes, and skills. It provided the assessment and instant feedback required in the competency-based medical education (CBME) learning process | **Education**  (LGBTQ+ education and care) | 1.Games encouraged student participation and benefited gender knowledge transmission. | 1.The need for teachers to improve their teaching competency to fully implement CBME using games.  2.Structural and systemic biases within the medical education system.  3.Potential resistance to integrating game-based teaching methods in traditional curricula. |
| [121] Yang 2021 | Medical students and teachers | 19 medical students and 2 teachers; Qualitative case-study methodology using in-depth interviews and thematic analysis | The study designed an LGBT+ Health and Medical Care course in a medical school, incorporating case-based teaching activities to promote gender competency. | The findings indicated that case-based teaching encouraged student participation and benefited gender knowledge transmission and transformation through competency learning | Case-based teaching successfully guided medical. It provided the assessment and instant feedback required in the competency-based medical education (CBME) learning process | **Education**  (LGBTQ+ education and care) | 1.Effective cases link theory to clinical practice and competency learning.  2.Experience sharing by LGBT+ individuals is highly effective.  3.High-quality narrative cases are crucial teaching materials to achieve LGBT+ competency and stories told by LGBT+ people were most effective strategy | Not applicable |
| [122] Salkind et al. 2019 | Medical students from a large London-based medical school. | 433 students before the session and 541 students after the session; Quantitative evaluation of compulsory teaching program | The intervention included a compulsory teaching program on LGBT+ health, aiming to increase student confidence in using appropriate terminology and performing clinical assessments on LGBT+ patients | The teaching program significantly increased students' confidence in using appropriate terminology related to sexual orientation and gender identity, as well as in performing clinical assessments on LGBT+ patients. | Confidence in using appropriate terminology to describe sexual orientation increased from 62% to 93%, gender identity increased from 41% to 91% | **Education**  (LGBTQ+ education and care) | 1.Collaboration with the LGBT+ community in designing and delivering the teaching program.  2.Inclusion of real-life experiences through the visit of a transgender patient.  3.Higher confidence and better knowledge how to behave transgender | 1.Need for ongoing training and support for educators to effectively teach LGBT+ health topics. |
| [123] Kellett & Fitton 2017 | Nursing students and educators | Discussion about the broader context of nursing education and practice | The article proposes several curricular and structural-level interventions to increase the recognition of gender diversity in nursing education and practice. | The article aims to raise awareness about the problems inherent to transinvisibility and to propose interventions that may serve to gradually increase the recognition of gender diversity in the planning and delivery of nursing education and practice | The proposed interventions are intended to create a more inclusive and supportive environment for transgender clients and nurses, thereby improving the overall quality of nursing care. | **Education**  (LGBTQ+ education and care) | 1.Inclusive information systems.  2.Creation of gender-neutral and safe spaces.  3.Lobbying for inclusion of competencies that address care of transgender persons in accreditation standards and licensure examinations. | 1.The hidden curriculum in nursing education that perpetuates cisnormativity.  2.Lack of awareness and education about transgender health issues.  3.Structural and systemic biases within the nursing profession. |
| [124] Henriquez et al. 2019 | Undergraduate nursing students | Implementation of an unfolding LGBTQ family case study as a teaching approach | Unfolding LGBTQ family case study designed to improve nursing students' understanding of family dynamics and care for LGBTQ older adults | Facilitated student understanding of the unique health and social issues for LGBTQ older adults within a family context. Students gained knowledge concerning shifts in family structures and the nurses' role in encouraging inclusiveness and equitable access in healthcare settings. | Increased knowledge of family diversity and critical thought regarding the intersectionality of discrimination and aging | **Education**  (LGBTQ+ education and care) | 1.Addressing the challenges of aging for transgender individuals within the context of societal stigma.  2.Encouraging inclusiveness and equitable access in healthcare settings.  3. Enhances acceptance, skills, knowledge and gender-affirming communication techniques | 1.Challenges related to societal stigma and discrimination |
| [125] Marshall et al. 2023 | Primary care providers in Arkansas | 20 primary care providers; Qualitative study using semi-structured interviews. | Explore the experiences and perceptions of primary care providers regarding the facilitators and barriers to providing affirming care for transgender patients | Providers identified several facilitators and barriers to providing affirming care for transgender patients. Facilitators included supportive clinic environments and provider education, while barriers included lack of training, systemic issues, and personal biases. | The study highlighted the importance of creating supportive clinic environments and providing education to healthcare providers to improve affirming care for transgender patients. | **Education**  (LGBTQ+ education and care) | 1.Supportive clinic environments.  2.Provider education and training.  3.Access to resources and guidelines for transgender care. | 1.Lack of training and education on transgender health.  2.Systemic issues such as insurance and healthcare policies.  3.Personal biases and discomfort among providers. |
| [126] Rambarran et al. 2016 | General Practitioners (GPs) in Barbados | 10 GPs; Qualitative study using semi-structured interviews. | Conducted to explore the experiences and perspectives of GPs regarding LGBT patients | The study revealed scant discussions on sexual health, practices, and identity among GPs. There were varied understandings of sexual and gender identity, and LGBT patients were often invisible with their specific healthcare needs not adequately addressed. | The study highlighted the need for enhanced GP training to improve LGBT patient care, addressing societal, professional, and methodological challenges to implementation | **Education**  (LGBTQ+ education and care) | 1.Enhanced GP training on LGBT health issues.  2.Increased awareness and understanding of sexual and gender identity.  3.Supportive clinic environments that promote inclusivity. | 1.Varied understandings of sexual and gender identity among GPs.  2.Invisibility of LGBT patients and their specific healthcare needs  3.Consultation timing and context were major challenges |
| [127] Sequeira et al. 2021 | Transgender youth aged 12 to 26 years receiving care in a multidisciplinary gender clinic | Transgender youth (n= 204); Quantitative survey | The study surveyed transgender youth to assess their interest in receiving gender-affirming care via telemedicine or through primary care with telehealth support | Almost half (47%) of the surveyed youth expressed interest in receiving gender care via telemedicine | Increased interest in telemedicine was seen among youth with lower perceived parental support | **Education**  (LGBTQ+ education and care) | 1.Telemedicine provides accessible and convenient care.  2.Primary care settings with telehealth support. | 1.Lack of telehealth infrastructure in primary care settings  2.Transgender youth with limited parental support are at  higher risk of experiencing mental health disparities and  barriers to accessing gender-affirming care |
| [128] Erdsiek et al. 2022 | Administration managers of German hospitals | 112 hospitals; Mixed-mode survey using pen-and-paper and online questionnaires | The survey examined measures and structures hospitals employ to address diversity | Diversity in their mission statements (57.1%) and quality management (59.9%). Working groups and diversity commissioners were less prevalent (15.2% each). Multilingual services were offered in admission and exit interviews (59.8%) and treatments or therapies (57.1%), but less so in meal plans (12.5%) | Practical measures were not widely implemented, and there was no overarching concept to address diversity comprehensively | **Institutions of care (medical) patient record** | 1.Inclusion of diversity considerations in mission statements and quality management.  2.Supportive clinic environments that promote inclusivity. | 1.Lack of financial resources  2.Lack of incentives from funding providers (49.1%).  3.Organizational difficulties |
| [129] Kassenärztliche Bundesvereinigung 2015 | Healthcare professionals participating in quality circles | Handbook for quality circles focused on gender-sensitive healthcare | Outlines the implementation of gender-sensitive healthcare practices within quality circles. It includes educational modules, practical tools, and guidelines for moderators to facilitate discussions and training on gender-specific aspects of patient care. | The aim is to improve the understanding and integration of gender-sensitive healthcare practices among healthcare professionals, thereby enhancing patient care and outcomes | The document emphasizes the importance of recognizing gender differences in healthcare and provides strategies for incorporating gender-sensitive practices into everyday clinical work | **Institutions of care (medical) patient record** | 1.Comprehensive educational modules for practitioners and practical tools for quality circle moderators. | 1.Lack of awareness and education about gender-sensitive healthcare among healthcare professionals.  2.Organizational challenges in implementing gender-sensitive practices.  3.Limited resources and support for continuous training and development in gender-sensitive healthcare. |
| [130] Deutsch et al. 2013 | Expert clinicians and medical information technology specialists | Recommendations from the WPATH EMR Working Group. | WPATH Executive Committee convened an Electronic Medical Records Working Group to make recommendations for developers, vendors, and users of EHR systems with respect to transgender patients | The recommendations aim to ensure that EHR systems can accommodate the unique needs of transgender patients, such as recording chosen names and gender identities that differ from legally designated names and sexes, and managing sex-specific health information | The implementation of these recommendations is intended to improve the quality of care for transgender patients by ensuring that their health records accurately reflect their identities and health needs | **Institutions of care (medical) patient record** | 1.Inclusion of fields for chosen names and gender identities in EHR systems.  2.Ability to record and manage sex-specific health information  3.Providers and clinic staff notify of a patient’s preferred name and/or pronoun | 1.Lack of awareness and understanding of transgender health  2.Resistance to change and implementation of new practices within healthcare organizations. |
| [131] Grasso et al. 2019 | Healthcare providers and administrators involved in the implementation of electronic health records (EHR) systems | Recommendations based on best practices and evidence from a federally qualified health center | Recommendations for planning and implementing high-quality sexual orientation and gender identity (SO/GI) data collection in primary care and other healthcare practices | The recommendations aim to improve access to and quality of care for LGBTQ patients by ensuring that SO/GI data is accurately collected and integrated into EHR systems | Implementing SO/GI data collection in EHR systems is expected to enhance the identification of health disparities, monitor risk behaviors, and assess progress toward reducing disparities among LGBTQ populations | **Institutions of care (medical) patient record** | 1.Training and education for healthcare providers on the importance of SO/GI data.  2.Supportive clinic environments  3.Patients should feel comfortable | 1.Lack of awareness and understanding of the importance of SO/GI data among healthcare providers.  2.Technical challenges  3.Resistance to change and implementation of new practices within healthcare organizations |
| [132] Lindsay et al. 2022 | Pediatric rehabilitation healthcare providers | Healthcare providers (19 women, 3 men, 1 transgender man); Qualitative needs assessment | Semi-structured interviews were conducted to explore the perceived challenges in providing gender-sensitive care. | The study identified several challenges in providing gender-sensitive care, including lack of training and experience, gender differences and stereotypes, binary documentation, complexity of gender identity, and the gender of the clinician | The findings highlighted the need for further training and systemic support to improve gender-sensitive care in pediatric rehabilitation settings | **Institutions of care (medical) patient record** | 1.Increased training and education on gender-sensitive care.  2.Supportive clinic environments that promote inclusivity.  3.Development of guidelines and best practices for gender-sensitive care. | 1.Lack of training and experience among healthcare providers.  2.Binary documentation systems that may lead to misgendering.  3.Complexity of gender identity and related stereotypes. |
| [133] Martos et al. 2019 | Lesbian, gay, and bisexual (LGB) individuals in the United States | 1,534 LGB individuals; Nationally-representative survey analyzing data across three age cohorts | The study examined the utilization of LGBT-specific clinics and providers among LGB individuals. It analyzed factors influencing past utilization and interest in future use of these healthcare services. | The study found that only 13% of LGB individuals had utilized LGBT-specific clinics and providers, but 52% expressed interest in using them in the future. Utilization was associated with a better overall sense of health, and interest in future use was higher among younger, Black LGB individuals and those with lower incomes | The study results emphasized the need for LGBT-specific clinics and providers to reach a more diverse sexual minority populace. | **Institutions of care (medical) patient record** | 1.Supportive clinic environments that promote inclusivity. | 1.Limited access to LGBT-specific clinics  2.Systemic barriers and lack of awareness about the availability of LGBT-specific healthcare services  3.LGBT-specific clinics and providers suggests a large disconnect between the kind of healthcare that many LGBs would like to have and what they may have access to |
